# Small RNAs mediate transgenerational inheritance of genome-wide *trans*-acting epialleles in maize

**DOI:** 10.1186/s13059-022-02614-0

**Published:** 2022-02-09

**Authors:** Shuai Cao, Longfei Wang, Tongwen Han, Wenxue Ye, Yang Liu, Yi Sun, Stephen P. Moose, Qingxin Song, Z. Jeffrey Chen

**Affiliations:** 1 State Key Laboratory of Crop Genetics and Germplasm Enhancement, Nanjing Agricultural University, 1 Weigang Road, Nanjing, 210095 China; 2 College of Life Sciences, Shanxi Agricultural University, Taigu, 030801 China; 3 Department of Crop Sciences, University of Illinois, Urbana, IL 61801 USA; 4 Department of Molecular Biosciences, The University of Texas at Austin, Austin, TX 78712 USA

**Keywords:** Hybrid, Small RNA, DNA methylation, Epialleles, Paramutation, Transgenerational inheritance, Backcrossing, Maize

## Abstract

**Background:**

Hybridization and backcrossing are commonly used in animal and plant breeding to induce heritable variation including epigenetic changes such as paramutation. However, the molecular basis for hybrid-induced epigenetic memory remains elusive.

**Results:**

Here, we report that hybridization between the inbred parents B73 and Mo17 induces *trans*-acting hypermethylation and hypomethylation at thousands of loci; several hundreds (~ 3%) are transmitted through six backcrossing and three selfing generations. Notably, many transgenerational methylation patterns resemble epialleles of the nonrecurrent parent, despite > 99% of overall genomic loci are converted to the recurrent parent. These epialleles depend on 24-nt siRNAs, which are eliminated in the isogenic hybrid Mo17xB73:*mop1-1* that is defective in siRNA biogenesis. This phenomenon resembles paramutation-like events and occurs in both intraspecific (Mo17xB73) and interspecific (W22xTeosinte) hybrid maize populations. Moreover, siRNA abundance and methylation levels of these epialleles can affect expression of their associated epigenes, many of which are related to stress responses.

**Conclusion:**

Divergent siRNAs between the hybridizing parents can induce *trans*-acting epialleles in the hybrids, while the induced epigenetic status is maintained for transgenerational inheritance during backcross and hybrid breeding, which alters epigene expression to enhance growth and adaptation. These genetic and epigenetic principles may apply broadly from plants to animals.

**Supplementary Information:**

The online version contains supplementary material available at 10.1186/s13059-022-02614-0.

## Background

Transgenerational epigenetic inheritance is the transmission of epigenetic states between generations without alteration of primary DNA sequence [1]. Epigenetic states including DNA methylation and histone modifications may be altered in response to internal (genomic) and external (environmental) signals or stresses in plants and animals [2, 3]. In animals, toxins and/or nutritional changes can cause alteration of epigenetic states for gene expression, leading to intergenerational variation (not necessary heritable) or transgenerational epigenetic inheritance [3]. Recent studies in *Caenorhabditis elegans* have shown that small interfering RNAs (siRNAs) are involved in neural gene expression and chemotaxis behavior in three generations [4] and in a long-term memory of avoidance to pathogens [5]. In plants, siRNAs can induce silencing [6] and RNA-directed DNA methylation (RdDM) to enforce epigenetic states [7]. The siRNA-mediated RdDM pathway is responsible for stress-induced activation of transposable elements (TEs) in *Arabidopsis* [8, 9] and rice [10, 11], which involves RdDM for transmission. In rice, stress-induced gene expression is the cause but not the effect of RdDM [10, 11].

Allelic interactions in the hybrid (heterozygous state) can induce allelic expression changes in the offspring, as reported in the epigenetic phenomenon known as paramutation in plants [12–15] and later in mice [3], although some paramutation-like events are related to parent-of-origin effects as observed in mice [16], flies [17], and worms [18]. Mechanisms for those *trans*-acting changes involve small RNAs and RdDM as shown in maize [19]. DNA methylation in plants occurs in CG, CHH, and CHG (H = A, T or C) [20]; CHH methylation is largely established through the RdDM pathway [21], involving biogenesis of siRNAs by the RNA polymerase Pol II homologs, Pol IV and Pol V, and a RNA-dependent RNA polymerase (RDR) [22, 23]. Paramutation in maize is disrupted in *mediator of paramutation1* (*mop1*), which encodes an RDR2-like protein [19]. In the maize *mop1-1* mutant with loss of function in *Mop1-B73*, CHH methylation levels are substantially reduced [24], and are accompanied by decrease of 24-nt siRNAs [25]. Moreover, most siRNA distribution differences between the hybridizing parents are also present in the F_1_ progeny [26]. A recent study in the *mop1-1* mutant also found increased recombination in chromosomal arms but reduced recombination in pericentromeric regions [27]. In *Arabidopsis thaliana* F_1_ hybrids DNA methylation differences are known as *trans*-chromosomal methylation (TCM) and demethylation (TCdM) [28]. These TCM and TCdM loci are associated with 24-nt siRNAs, which disappear in *A. thaliana* hybrids of the *nrpe1 nrpd1* mutants [29].

Transgenerational inheritance of DNA methylation has been observed in *A. thaliana* [30]. In maize, differential methylation between inbred lines is heritable, and differentially methylated regions can shift from one epiallele to the other, which are associated with 24-nt siRNAs, and are stably inherited in recombinant inbred lines [31]. In cotton allotetraploids that were formed over ~ 1.5 million years ago [32], subsets of hybridization-induced epialleles were maintained during evolution, selection, and domestication, suggesting long-term epigenetic inheritance [33]. However, mechanisms for inheritance of *trans*-acting epialleles remain poorly understood.

Here, we investigated transgenerational inheritance of genome-wide *trans*-acting epialleles derived from the reciprocal F_1_ hybrids (BxM and MxB) (by convention, the maternal parent is listed first in a genetic cross) between the maize inbred lines B73 (B) and Mo17 (M). The *trans*-acting differentially methylated regions (DMRs) between F_1_ and two parents were transmitted through six backcrossing followed by three selfing generations; they are called transgenerational DMRs (tgDMRs) or epialleles. Notably, many epialleles resembled those from the nonrecurrent parent, despite their genomic sequences were converted to the recurrent parent. Transgenerational inheritance of epialleles was also observed in the interspecific backcross progeny derived from W22 and teosinte. Furthermore, initiation of *trans*-acting methylation loci in F_1_ and transmission of epialleles during breeding depended on siRNAs and the RdDM pathway. Finally, some heritable epialleles are associated with expression variation of stress-responsive genes in the F_1_ and backcross-selfing progeny. Collectively, we demonstrate a role of siRNA-mediated DNA methylation in genome-wide transgenerational inheritance of epialleles that can influence gene expression and phenotypes in the offspring.

## Results

### Methylation changes induced in F_1_ hybrids were heritable over multiple generations

**Fig. 1:**
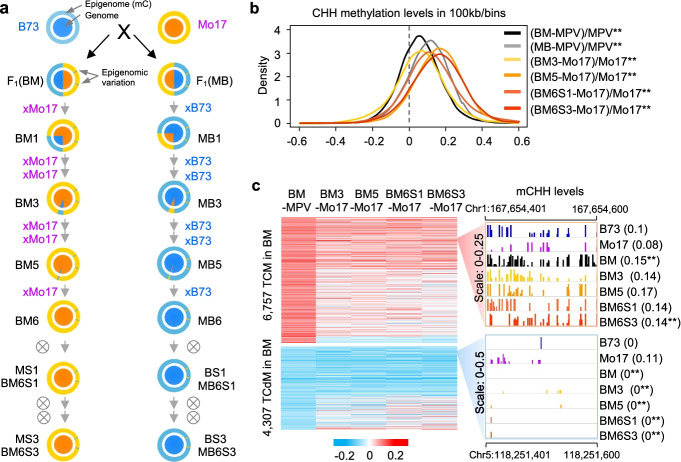
Hybrid-Induced DNA methylation changes are transgenerational through multiple generations of backcrossing and selfing. **a** Schemes for producing genetic materials. F_1_ hybrid (BM) was made between B73 (maternal parent) and Mo17 (as pollen donor) and backcrossed consecutively with Mo17 (recurrent parent) to produce backcross lines from BM1 (F_1_BC1) to BM5 (F_1_BC5); BM6 (F_1_BC6) was subsequently self-pollinated as BM6S1 for three generations to produce BM6S3. Likewise, the reciprocal F_1_ hybrid MB underwent a similar backcrossing scheme with B73 pollen to produced MB1 (F_1_BC1), MB3 (F_1_BC3), MB5 (F_1_BC5), and MB6 (F_1_BC6) that was self-pollinated to produce MB6S1-MB6S3. Large circles indicate epigenome (methylome) of B73 (light blue) and Mo17 (yellow), and small circles indicate nuclear genomes of B73 (blue) and Mo17 (orange). **b** Kernel density distribution of relative methylation changes (*y*-axis) in the F_1_ hybrids compared to the mid-parent value (MPV) and in the backcross lines compared to their recurrent parents. The density is estimated by the difference (I-II) divided by II (with a 100-kb bin window). Line colors indicate BM (black), MB (gray), BM3 (light yellow), BM5 (yellow), BM6S1 (orange), and BM6S3 (red). Double asterisks indicate statistical significance (*P* < 0.01, two-sided Wilcoxon signed-rank test). **c** Heatmaps show CHH methylation changes in the TCM and TCdM loci (left) and two examples (right). Methylation levels were increased in 6757 TCM loci (upper panel) and decreased in 4307 TCdM loci (lower panel) in all backcross and selfing lines examined. Examples are Chr1: 167,654,401-167,654,600 (TCM, upper panel) and Chr5: 118,251,401-118,251,600 (TCdM, lower panel). Numbers in parentheses indicate average methylation levels for the DMRs. Double asterisks indicate statistically significant changes between a F_1_ hybrid and MPV or between a backcross line and its recurrent parent (*P* < 0.05, one-way ANOVA test)

To test epigenetic inheritance, we generated the F_1_ hybrid (Mo17xB73, MB) and the reciprocal hybrid (B73xMo17, BM) (Fig. 1a). The MB hybrid was backcrossed to B73 for six generations (MB1 to MB6), followed by self-pollination for three generations (MB6S1 to MB6S3). Likewise, the reciprocal hybrid (BM) was backcrossed to Mo17 for six generations (BM1 to BM6), followed by self-pollination for three generations (BM6S1 to BM6S3). MB6S3 and BM6S3 populations (55 plants each) were used for genotyping analysis using 20 indel markers (Additional file 1: Fig. S1a). We found that most plants in each population were homozygous for the recurrent parent alleles. Nine of the BM6S3 lines and six of the MB6S3 lines were further genotyped using MaizeSNP6K array [34] (Additional file 2: Table S1). The results confirmed 92.0% or higher levels of homozygosity to their respective recurrent parents (Additional file 1: Fig. S1b and Additional file 6: Dataset S1a), including BM6S3-2 (99.4% homozygosity) and MB6S3-3 (99.1%), which represented BM6S3 and MB6S3, respectively, and were used for further analysis.

Single-base resolution DNA methylomes were generated from 12 genotypes, including inbred parents B73 and Mo17 and their reciprocal F_1_ crosses (MB and BM), four from 3rd and 5th backcross generations (MB3, MB5, BM3, and BM5), and four reciprocal backcross-selfing lines (BM6S1, BM6S3, MB6S1, and MB6S3) (Additional file 3: Table S2). Sequencing reads of each line with two biological replicates were mapped to the combined genome of B73 and Mo17, and the ratio for the uniquely mapped reads was 1:1 (B73:Mo17) in the F_1_ hybrids (Additional file 1: Fig. S1c and Additional file 6: Dataset S1b). This suggests that these materials are suitable for testing DNA methylation changes during multiple generations of backcrossing and selfing. To avoid excluding duplicate reads, we employed three approaches. Firstly, sequencing reads from B73, MB3, MB5, and MB6S1 and MB6S3 lines were mapped onto the B73 RefGen_v4 genome [35] to detect methylation changes during backcross to B73. Secondly, sequencing reads from Mo17, BM3, BM5, BM6S1, and BM6S3 lines were mapped onto the pseudo-genome of Mo17 [36], in which the B73 reference was replaced with corresponding Mo17 SNPs to detect methylation changes among Mo17 backcross lines. Finally, clean reads of reciprocal F_1_ hybrids (BM and MB) were mapped onto B73 RefGen_v4 genome to detect methylation changes induced by hybridization. To minimize the effect of SNPs between B73 and Mo17 (especially between C and T) on DNA methylation analysis, overlapped cytosines between all lines and present in both biological replicates were retained for analysis [33].

These analyses found substantial changes in CHH methylation levels (in 100-kb/bins) in the hybrids or backcross lines (Fig. 1b and Additional file 1: Fig. S1, d-h and Additional file 6: Dataset S1, c-d), while CHG and CG methylation levels did not display obvious changes and could be used as internal controls. For further analysis, we focused on changes in the CHH methylation. In reciprocal F_1_ hybrids, CHH methylation levels were increased relative to the mid-parent value (MPV, average of the two parents) in BM or MB hybrids (Fig. 1b and Additional file 6: Dataset S1e). We tested if the hyper and hypo DMRs induced in the F_1_ hybrids can transmit through backcross breeding. Principal component analysis (PCA) of DMRs showed clear separation between B73 with all backcross lines and Mo17 with its backcross lines, with the reciprocal hybrids (MB and BM) in the middle (PC1; Additional file 1: Fig. S2a), suggesting potential inheritance of DMR patterns in backcross generations. As expected, overall methylation changes were positively correlated between BM F_1_ hybrid and Mo17 backcross lines (Pearson’s correlation coefficient: *r* = 0.2–0.3, *P* < 2.2e^−16^) or between MB F_1_ hybrid and B73 backcross lines (*r* = 0.25–0.3, *P* < 2.2e^−16^) (Additional file 1: Fig. S2b and Additional file 7: Dataset S2a). Percentage of variation was higher in the former than in the latter (Additional file 1: Fig. S2a), suggesting a parent-of-origin effect on methylation inheritance, as observed in *A. thaliana* hybrids [37].

We adopted the terms TCM and TCdM to describe *trans*-acting (chromosomal) methylation (TCM or hyper) and demethylation (TCdM or hypo) events [28], respectively, in which the methylation level of one parental allele in the F_1_ progeny is *trans*-altered to resemble the methylation status of the other parental allele. We identified 6757 TCM DMRs and 4037 TCdM DMRs in the BM hybrid and 8723 TCM DMRs and 5212 TCdM DMRs in the reciprocal MB hybrid (Additional file 1: Fig. S2c and Additional file 7: Dataset S2b). The results of more TCM DMRs than TCdM DMRs were consistent with increased overall methylation levels in both MB and BM hybrids (Fig. 1b and Additional file 6: Dataset S1e). Only a small proportion of TCM DMR (704) and TCdM DMR (652) loci was shared between the two hybrids (*P* = 0 in TCM or TCdM DMRs, hypergeometric test) (Additional file 1: Fig. S2e), suggesting methylation increase or decrease of these alleles in both hybrids, while the majority of them exhibit parent-of-origin effects.

We tabulated numbers of DMRs in all generations including F_1_ (TCdM and TCM), BM5 and MB5, MB6S1 and BM6S1, and MB6S3 and BM6S3 populations tested (Table 1). Approximately ~ 7.5% TCdM (hypo) DMRs and ~ 2.4% TCM (hyper) DMRs in the BM hybrid were also identified as hypo DMRs (Additional file 1: Fig. S3b and Additional file 7: Dataset S2d) and hyper DMRs (Additional file 1: Fig. S3c), respectively, in each of Mo17 backcross generation (BM6S1 or BM6S3). The same trend was observed in the B73 backcross lines, including ~ 11% TCdM DMRs (Additional file 1: Fig. S3d and Additional file 7: Dataset S2e) and ~ 2.8% TCM DMRs (Additional file 1: Fig. S3e) in the MB hybrid, which remained hypo- and hypermethylation in respective B73 backcross generation (MB6S1 or MB6S3). As examples, 102 TCM (hyper) DMRs in the BM hybrid remained as hyper DMRs in BM6S3 and also showed increased methylation levels in all other Mo17 backcross generations (Table 1), while 225 TCdM (hypo) DMRs remained as hypo DMRs in BM6S3 with decreased methylation levels in all other backcross lines. These TCM and TCdM DMRs that were induced by hybridization and transmitted to the last backcross-selfing generation (BM6S3 or MB6S3) were called transgenerational DMRs (tgDMRs) or epialleles. For example, the TCM region (Chr1: 167,654,401–167,654,600) in BM remained to be methylated in all Mo17 backcross lines tested, including BM3, BM5, BM6S1, and BM6S3 (Fig. 1c), whereas the TCdM region (Chr5: 118,251,401–118,251,600) in BM had low methylation levels among all generations of Mo17 backcross lines (Fig. 1c).

**Table 1:** Hybridization-induced CHH DMRs and their transgenerational (tgDMR) inheritance in maize

**DMRs (in F**_**1**_**)**	**F**_**1**_ **(BM)**	**No. of TCM or TCdM DMRs present in each Mo17 backcross line**				**tgDMRs present in BM6S3 line**	**tgDMRs with SNPs**	**tgDMRs with methylation levels similar to that of the nonrecurrent parent Mo17**
		**BM3**	**BM5**	**BM6S1**	**BM6S3**			
Hyper (TCM)	8417	218	270	271	188	102	19	2
Hypo (TCdM)	3561	388	301	241	307	225	60	116
**DMRs (in F**_**1**_**)**	**F**_**1**_ **(MB)**	**No. of TCM or TCdM DMRs present in each B73 backcross line**				**tgDMRs present in MB6S3 line**	**tgDMRs with SNPs**	**tgDMRs with methylation levels similar to that of the nonrecurrent parent B73**
		**MB3**	**MB5**	**MB6S1**	**MB6S3**			
Hyper (TCM)	6757	269	180	274	221	99	19	27
Hypo (TCdM)	4037	433	497	432	456	363	35	155

Interestingly, although TCM DMRs (6757 in the MB hybrid and 8723 in the BM hybrid) were higher than TCdM DMRs (4037 in the BM hybrid, and 5212 in the MB hybrid) (Additional file 1: Fig. S2c and Additional file 7: Dataset S2b), 3-6-fold or more TCdM DMRs than TCM DMRs (5.5% vs. 1.5% in the BM hybrid and 6.9% vs. 1.1% in the MB hybrid) were transmitted in the Mo17 and B73 backcross-selfing populations, respectively.

These 327 and 462 tgDMRs in Mo17 and B73 backcross lines, respectively (Table 1), were significantly enriched in 5′ (12.8% and 15.3% in Mo17 and B73 backcross lines, respectively) and 3′ (12.2% and 7.6% in Mo17 and B73 backcross lines, respectively) regions flanking the coding sequence (Additional file 1: Fig. S4a and Additional file 7: Dataset S2h) (*P* = 0, *chi*-square test). To determine a possible effect of structural variation (SV) on tgDMRs between B73 and Mo17 genomes, we identified 175,849 SV (Additional file 1: Fig. S4b and Additional file 7: Dataset S2i) and found that 2.1% (7/327) tgDMRs in Mo17 backcross-selfing lines and 3.9% (18/462) tgDMRs in B73 backcross-selfing lines overlapped with SV breakpoints, which was higher than 1.3% (58,721/4,645,542) at the whole genome level (Additional file 1: Fig. S4c and Additional file 7: Dataset S2j) (*P* = 0, *chi*-square test). Furthermore, we found 2.1% (949/44,799) of all DMRs between B73 and Mo17 overlapped with SV (Additional file 1: Fig. S4d), suggesting a contribution of SV to methylation changes in flanking loci [38]. The tgDMRs with SV (7 in Mo17 backcross-selfing lines and 18 in B73 backcross-selfing lines) showed higher levels of methylation changes than tgDMRs without SV (*P* < 1.8e−3, Wilcoxon signed-rank test) (Additional file 1: Fig. S4d). Although the total number of tgDMRs with SV was small, these SV-associated DMRs tended to be transgenerationally inherited.

We further investigated chromatin status of tgDMRs using published epigenetic datasets, including Dnase-seq [39], ATAC-seq, and ChIP-seq of histone modifications (H3K27me3, H3K36me3, H3K56ac, H3K4me3, H3K4me1) [40] (NCBI accession no. PRJNA381532, PRJNA527732). We found that these tgDMRs (327 in BM6S3 and 462 in MB6S3) were associated with reduced chromatin accessibility (Additional file 1: Fig. S4f and Additional file 7: Dataset S2k) and depleted with H3K36me3, H3K56ac, H3K4me3, and H3K4me1 histone marks and H2A.Z (Additional file 1: Fig. S4g and Additional file 7: Dataset S2k). In contrast, H3K27me3, a repressive mark for gene expression, overlapped with these DMRs (Additional file 1: Fig. S4g and Additional file 7: Dataset S2k). These results indicate association of tgDMRs with chromatin inaccessibility and void of open chromatin sites.

### Transgenerational DNA methylation changes resembled the nonrecurrent parent

**Fig. 2:**
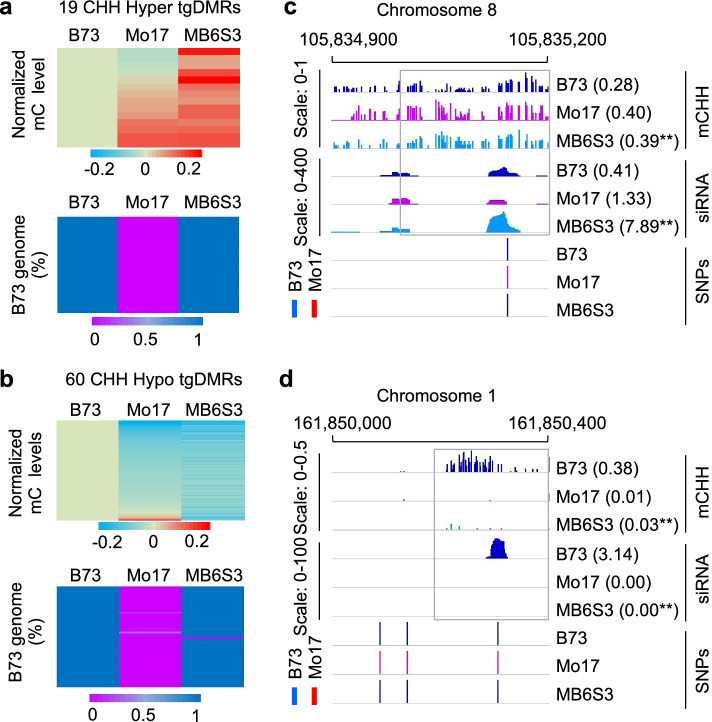
Transgenerational DNA methylation changes resembled the nonrecurrent parent. **a**, **b** Inheritance of 19 hyper allelic tgDMRs (**a**) and 60 hypo allelic tgDMRs (**b**) in the MB6S3 line (upper panels). Genomic regions in corresponding DMRs were converted to the recurrent parent B73 (lower panels). The percentage was estimated by the average B73/Mo17 allelic ratio in each DMR using SNPs of all loci. **c**, **d** Examples of Chr8: 105,835,001–105,835,200 (**c**) and Chr1: 161,850,201–161,850,400 (**d**) displaying high (**c**) and low (**d**) methylation and siRNA levels in both MB6S3 and donor parent Mo17. Scales are 0–1 or 0.5 and 0–400 or 100 for normalized methylation and siRNA levels, respectively, in each DMR. Numbers represent average levels of DMR methylation and siRNA expression (RPTM). Double asterisks indicate a significant change between MB6S3 and the recurrent parent B73 (*P* < 0.05, one-way ANOVA test for methylation and two-tailed Student’s *t*-test for siRNA expression)

Among all tgDMRs in BM6S3 and MB6S3 populations, there were allelic tgDMRs each with SNPs that could discriminate between B73 and Mo17 alleles, including 38 hyper (19 in MB6S3 and 19 in BM6S3) and 95 hypo (60 in MB6S3 and 35 in BM6S3) tgDMRs (Table 1, Fig. 2a, b, and Additional file 1: Fig. S3f, h and Additional file 7: Dataset S2, f-g). Notably, all allelic tgDMRs with one exception were genetically homozygous to the recurrent parent in the last backcross-selfing generation. Only the locus (Chr9:141101601-141101800) had residual B73 sequence in BM6S3.

Inheritance of these tgDMRs may suggest that *trans*-acting methylation changes in the F_1_ hybrids induced by the nonrecurrent parent were heritable during meiosis. As predicted, either hyper or hypo tgDMRs in MB6S3 (backcrossed to B73) resembled the high or low methylation levels of the nonrecurrent parent Mo17 (Fig. 2, a and b, upper panel and Additional file 7: Dataset S2f), despite > 99% overall genomic fragments of these DMRs in the MB6S3 were converted to the recurrent parent B73 (Fig. 2, a and b, bottom panel). For example, methylation levels of the hyper tgDMR (Chr8: 105,835,001-200) were high in both MB6S3 and Mo17 (Fig. 2c). Likewise, the hypo tgDMR (Chr1: 161,850,201-400) had low methylation levels in both MB6S3 and Mo17 (Fig. 2d). The same trend was observed in the reciprocal BM6S3 line (backcrossed to Mo17) (Additional file 1: Fig. S3f, upper panel and Additional file 1: Fig. S3h, upper panel and Additional file 7: Dataset S2g), which resembled high and low levels of the nonrecurrent parent B73, despite over 99% genomic regions of these DMRs in the BM6S3 were converted to the recurrent parent Mo17 (Additional file 1: Fig. S3, f and h, bottom panel). For example, the hyper tgDMR (Chr3: 181,013,201-18,101,3400) showed high methylation levels in both BM6S3 and B73 (Additional file 1: Fig. S3g), while the hypo tgDMR (Chr1: 227,434,601-227,434,800) displayed low methylation levels in both BM6S3 and B73 (Additional file 1: Fig. S3i). Among tgDMRs in Mo17 and B73 backcross lines (Table 1 and Additional file 4: Table S3), 36.1% (118/327) and 39.0% (182/462) that originated from their corresponding nonrecurrent parents in the F_1_ were transmitted to the BM6S3 and MB6S3 populations, respectively, after six backcross and three selfing generations (Table 1). This proportion (36-39%) of tgDMR inheritance was much higher than ~ 1.0% (44,798 DMRs out of 4,645,542 total genomic bins) in the whole genome level (*P* = 0, *chi*-square test).

### Initiation and maintenance of trans-acting tgDMRs involve 24-nt siRNAs

**Fig. 3:**
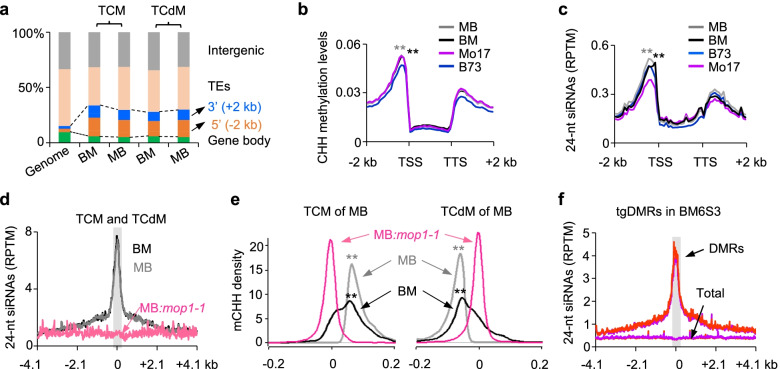
Initiation of TCM and TCdM loci and inheritance of tgDMRs depend on siRNAs. **a** Fraction of TCM or TCdM DMRs between F_1_ hybrids and the mid-parent value (MPV) in different genomic features, including gene body, flanking regions of 5′ (− 2 kb to the transcription start site, TSS) and 3′ (transcription termination site, TTS, to + 2 kb), TEs, and intergenic regions excluding TEs in MB, BM, and whole genome (genome). **b**, **c** Distribution (*y*-axis) of CHH methylation (**b**) and 24-nt siRNA (**c**) (reads per ten million, RPTM) levels in the genic regions of the F_1_ hybrids and their parents. Double asterisks indicate statistical significance (*P* < 0.01, two-sided Wilcoxon signed-rank test). **d** Levels of 24-nt siRNAs (RPTM, *y*-axis) in the MB (black), BM (gray), and MB:*mop1* (red) hybrid lines. Location of siRNA loci relative to the center (light grey) of TCM and TCdM regions is shown in *x*-axis. **e** Density (*y*-axis) of the methylation difference between the hybrids and their MPV among TCM (left panel) and TCdM (right panel) loci in the BM (black), MB (gray), and MB:*mop1-1* (pink). Scale of changes (0 = no change) is shown in *x*-axis. Double asterisks indicate statistical significance (*P* < 0.01, two-sided Wilcoxon signed-rank test) of the methylation difference between the MB:*mop1-1* and BM (black) or MB (grey). **f** Levels of 24-nt siRNAs (RPKM, *y*-axis) in BM6S3 (red) and its recurrent parent Mo17 (violet). Location of siRNA loci relative to the center (light grey) of transgenerational DMRs is shown in *x*-axis. “Total” represents siRNA expression of all windows of the whole genome

This transgenerational inheritance may require *trans*-acting factor(s) to initiate and maintain these epigenetic states, which can involve siRNAs and RdDM [20, 41]. To dissect these factors, we analyzed distribution of TCM and TCdM DMRs among genomic features and found that they were located more in the 5′ (15% and 9% in BM and MB hybrids, respectively) and 3′ (11% and 6% in BM and MB hybrids, respectively) regions flanking the coding sequence than the genome-wide average (Fig. 3a and Additional file 8: Dataset S3a). The loci in the 5′ region correlated with increased methylation levels (Fig. 3b and Additional file 8: Dataset S3b) (*P* < 7.6e^−19^ in BM and *P* < 2.0e^−12^ in MB, Wilcoxon signed-rank test) and high levels of 24-nt siRNAs (Fig. 3c and Additional file 8: Dataset S3c) (*P* < 1.6e^−5^ in BM and *P* < 3.4e^−29^ in MB, Wilcoxon signed-rank test). Other siRNAs such as 21/22-nt siRNAs may also be involved [42]. Indeed, distribution patterns of 21/22-nt siRNAs are consistent with those of 24-nt siRNAs (Additional file 1: Fig. S4e and Additional file 7: Dataset S2k), as these noncanonical siRNAs may involve silencing of TEs [42]. Those 24-nt siRNAs were present in 37.1% (BM) and 34.2% (MB) of hyper DMRs and 29.6% (BM) and 33.1% (MB) of hypo DMRs (Additional file 1: Fig. S5a and Additional file 8: Dataset S3d), which were significantly higher than the genome average (6.8% in BM and 6.5% in MB) (*P* < 0.05, Student’s *t*-test). These data suggest that 24-nt siRNAs correlate with *trans*-acting loci in the reciprocal hybrids.

In maize, *MOP1*, an ortholog of *RDR2*, is involved in biogenesis of 24-nt siRNAs and paramutation [19]. We generated and validated the Mo17XB73:*mop1-1* hybrid lines to test a role of small RNAs and RdDM in tgDRMs during backcrossing. The reciprocal hybrid (B73XMo17:*mop1-1*) was not used for this study, which might exclude some changes. However, in *Arabidopsis*, the same set of 24-nt siRNA and CHH methylation loci was lost in the reciprocal crosses ColXC24:*nrpd1/nrpe1* and C24XCol:*nrpd1/nrpe1* [29], suggesting similar siRNA and RdDM changes in both directions in vegetative tissues (leaves), except in the seeds, as observed in *Arabidopsis* [43, 44]. We found that the abundance of 24-nt siRNAs was substantially reduced in the hybrid MB:*mop1-1*, as in the B73:*mop1-1* and Mo17:*mop1-1* parents (Additional file 1: Fig. S5, b-d and Additional file 8: Dataset S3e) [26]. Moreover, these 24-nt siRNAs of all corresponding TCM and TCdM loci in the MB hybrid were eliminated in the MB:*mop1-1* hybrid (Fig. 3d and Additional file 8: Dataset S3g), suggesting positive association of siRNAs with *trans*-acting methylation.

We then investigated whether 24-nt siRNAs are required to initiate *trans*-acting methylation changes in maize F_1_ hybrids. Methylome-seq libraries were made from B73:*mop1-1*, Mo17:*mop1-1* and their hybrid MB:*mop1-1* each with two biological replicates. Using the same bioinformatic pipeline, we found that methylation levels of CHG and CHH, except for CG, were substantially reduced in all *mop1-1* mutants (Additional file 1: Fig. S5, e-g and Additional file 8: Dataset S3f) [27]. Loss of CHH DNA methylation in these mutants occurred in the 5′ and 3′ regions flanking the coding sequence in the MB:*mop1-1* hybrid and its parents (Additional file 1: Fig. S5e and Additional file 8: Dataset S3f), and the methylation levels were evenly reduced in the MB:*mop1-1* hybrid (Additional file 1: Fig. S5h and Additional file 8: Dataset S3i) (*P* = 0, Wilcoxon signed-rank test). Notably, loss of CHH methylation in the MB:*mop1-1* hybrid overlapped with increased CHH methylation of TCM loci and decreased CHH methylation of TCdM loci in MB (Fig. 3e and Additional file 8: Dataset S3h) or BM (Additional file 1: Fig. S5i and Additional file 8: Dataset S3j) F_1_ hybrids (*P* < 5.02e^−44^, Wilcoxon signed-rank test). These data may indicate that 24-nt siRNAs are required to establish these loci in the hybrids. Alternatively, maintenance of these methylation loci requires 24-nt siRNAs.

The presence of siRNAs in the tgDMRs of BM6S3 and MB6S3 lines (Fig. 2), similar to their respective nonrecurrent parents, suggests that these siRNAs are involved in maintaining these loci during backcross and selfing. To test this, we analyzed 24-nt siRNA abundance and found sharp peaks of the siRNA abundance of tgDMRs in BM6S3 (Fig. 3f and Additional file 8: Dataset S3k) and MB6S3 (Additional file 1: Fig. S5j and Additional file 8: Dataset S3l) lines, relative to the genome average. This suggests persistent presence of these siRNAs in maintaining tgDMRs during backcross and selfing. Moreover, methylation changes in these tgDMRs correlated with loss of siRNAs in the BM:*mop1-1* hybrid (*P* < 3.27e^−12^, Wilcoxon signed-rank test) (Additional file 1: Fig. S5, k-l and Additional file 8: Dataset S3, m-n). It is likely that a low methylation level in the TCdM loci may prevent from transcription by PolIV to generate 24-nt siRNAs. Alternatively, 24-nt siRNAs may dilute in tgDMRs during meiosis through backcrossing and selfing generations. Together, these results indicate that canonical RdDM pathway establishes TCM and TCdM loci in the F_1_ hybrids and can maintain them for six backcross and three selfing generations in maize.

**Fig. 4:**
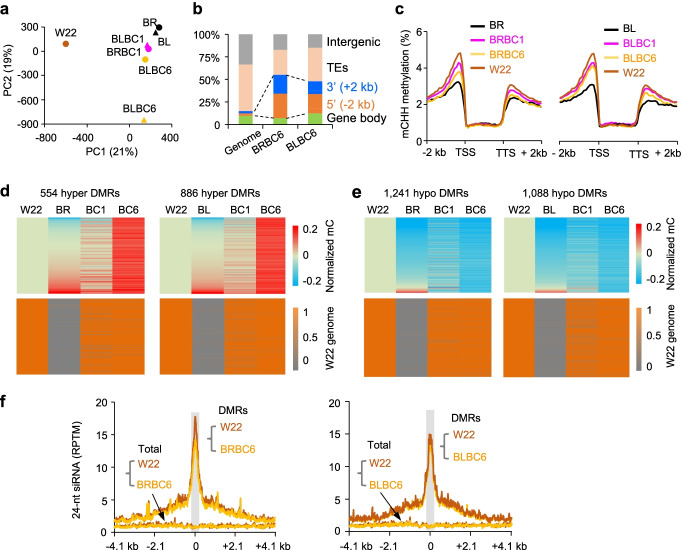
Transgenerational DNA methylation in backcross lines derived from maize W22 and teosinte. **a** Principal component analysis (PCA) of CHH methylation levels with the overall variance explained by PC1 (*x*-axis) and PC2 (*y*-axis). Genotypes include W22 (brown dot), BR (Bravo: black dot), BL (Blanco: black triangle), BRBC1 (W22XBR BC1, pink dot), BRBC6 (W22XBR BC6, yellow dot), BLBC1 (W22XBL BC1, pink triangle), and BLBC6 (W22XBL BC6, yellow triangle). **b** Fraction of CHH DMRs between BRBC (left) or BLBC (right) line and W22 in different genomic features, including gene body (light green) and flanking regions of 5′ (orange, − 2 kb to transcription start site, TSS) and 3′ (blue, transcription termination site, TTS, to + 2 kb), TEs (pink), and intergenic regions (grey) excluding TEs. **c** Distribution (*y*-axis) of CHH methylation in the genic regions of W22 backcross lines, W22, and teosinte. Line colors indicate BR (orange), BRBC1 (pink), BRBC6 (yellow), and W22 (black), left panel, and BL (orange), BLBC1 (pink), BLBC6 (yellow), and W22 (black), right panel. **d**, **e** Hyper DMRs (**d**) of 554 in BRBC6 (left panel) and 866 in BLBC6 (right panel) lines and hypo DMRs (**e**) of 1241 in BRBC6 (left panel) and 1088 in BLBC6 (right panel) lines. Genomic regions in corresponding DMRs were converted to the recurrent parent W22 (lower panels), which was estimated by the average allelic ratio in each DMR using SNPs of all loci. The methylation level was normalized to that of W22 (0) in each DMR. **f** Levels of 24-nt siRNAs (RPTM, *y*-axis) in DMRs between W22 and backcross in W22 (brown), BRBC6 (yellow, left panel), and BLBC6 (yellow, right panel). Location of siRNA loci relative to the center (grey) is shown in *x*-axis. “Total” represents siRNA expression from all windows of the whole genome

This *trans*-acting methylation occurs not only in the intraspecific hybrids but also in the interspecific maize hybrids that were formed between the modern maize W22 and teosinte (*Z. may* L*.* ssp. *parviglumis*) accessions Bravo (BR) or Blanco (BL) and transmit to their backcross lines. Using the published data [45], we showed that all backcross lines including BRBC1, BRBC6, BLBC1, and BLBC6 were close to BL and BR parents in PCA plots of methylation levels (Fig. 4a and Additional file 9: Dataset S4a), suggesting that the methylation levels in the backcross lines depend on the nonrecurrent teosinte parents BR and BL. These *trans*-acting methylation changes were also enriched in the vicinity of genes (Fig. 4b), especially in the 5′ region flanking the coding sequence (Fig. 4c and Additional file 9: Dataset S4b). Using the DMRs with SNPs that are distinguishable between W22 and teosinte (BR or BL) alleles, we found high methylation levels in most hyper DMRs (Fig. 4d, upper panel and Additional file 9: Dataset S4c) and low methylation level in almost all hypo DMRs (Fig. 4e, upper panel and Additional file 9: Dataset S4d) in the BC6 lines, resembling the teosinte parent, while the genome was largely converted to the recurrent parent W22 (Fig. 4, d–e, bottom panel and Additional file 9: Dataset S4, c-d). The data reinforce the notion that *trans*-acting methylation by the nonrecurrent parent teosinte can be maintained for at least six backcross generations, which also correlated with 24-nt siRNA levels (Fig. 4f and Additional file 9: Dataset S4e). These data together support a common mechanism for siRNAs in initiation and transgenerational inheritance of *trans*-acting epialleles induced by inter- and intraspecific crosses in *Z. mays*.

### Roles of *trans*-acting epialleles of tgDMRs in gene expression and phenotypic variation

**Fig. 5:**
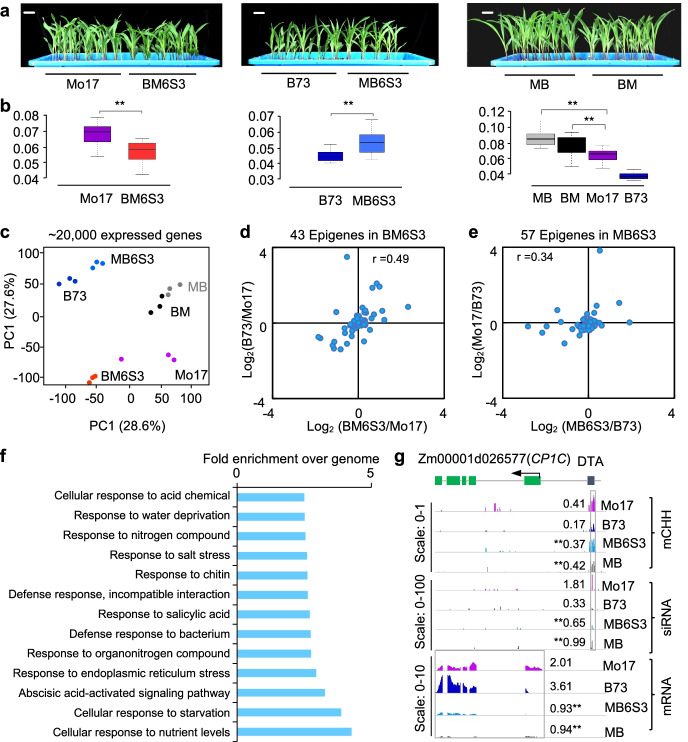
Transgenerational DMRs correlate with expression levels of epigenes. **a**, **b** Seedling phenotypes (**a**) and aboveground dry weight (**b**) at the same stage in Mo17 and BM6S3 (left), B73 and MB6S3 (middle), and MB and BM hybrids (right). Photos were taken at the same time with the same scale (bars = 10 mm). Double asterisks indicate statistical significance (*P* < 0.05 and *n* = 16 replicates, two-tailed Student’s *t*-test). **c** Principal component analysis (PCA) of transcriptome variation among six lines with the overall variance explained by PC1 (*x*-axis) and PC2 (*y*-axis). **d** Positive correlation (*r* = Pearson’s correlation coefficient) of expression level changes of 43 epigenes in MB6S3/B73 (*x*-axis) with those in Mo17/B73 (*y*-axis). **e** Positive correlation (*r* = Pearson’s correlation coefficient) of expression level changes of 57 epigenes in BM6S3/Mo17 (*x*-axis) with those in in B73/Mo17 (*y*-axis). **f** Gene Ontology (GO) overrepresentation of 97 tgDMRs-associated genes in MB6S3 and BM6S3 lines (*P* < 0.05 and fold enrichment > = 2.5). **g** An example of the epigene (*Zm00001d026577*), showing location of DTA transposon, mRNA, siRNA, and CHH methylation levels in Mo17, B73, MB6S3, and MB lines. Scales shown are 0–1 (CHH methylation), 0–100 (siRNA), and 0–10 (mRNA). Numbers indicate average methylation levels of DMR methylation, siRNA expression (RPTM), and gene expression (FPKM). Double asterisks indicate statistically significant changes between MB hybrid and MPV or between MB6S3 and its recurrent parent B73 (*P* < 0.05, one-way ANOVA test for methylation; two-tailed Student’s *t*-test for siRNA and gene expression)

As expected, both BM and MB hybrids exhibited seedling biomass heterosis (Fig. 5, a-b and Additional file 10: Dataset S5a), although grain weight was similar among six genotypes, including B73, Mo17, BM, MB, BM6S3, and MB6S3 (Additional file 1: Fig. S6a and Additional file 10: Dataset S5b). Biomass dry weight was higher in the MB6S3 than in the B73 but lower in BM6S3 than in Mo17 (Fig. 5a, b), despite > 99% overall genomic loci in BM6S3 and MB6S3 lines were converted to their corresponding recurrent parents. This result may suggest a role for these *trans*-acting DMRs in gene expression and phenotypic variation. Using principal component analysis (PCA) with ~ 20,000 expressed genes (EGs), we showed separation of overall gene expression between MB6S3 and B73 and between BM6S3 and Mo17, although MB6S3 and B73 were clearly divergent from BM6S3 and Mo17 (Fig. 5c). This difference of gene expression between the backcross-selfing line and its recurrent parent may suggest an effect of transgenerational methylation changes on gene expression. Here, a gene associated with tgDMR is named an epigene. Among 43 epigenes in BM6S3, log_2_-fold expression changes in BM6S3/Mo17 were positively correlated with those in the nonrecurrent parent B73 (*r* = 0.49, *P* = 1.86e^−4^) (Fig. 5d and Additional file 10: Dataset S5e). Moreover, among 57 epigenes in the reciprocal cross MB6S3, log_2_-fold expression changes in MB6S3/B73 were also positively correlated with those in the nonrecurrent parent Mo17 (*r* = 0.34, *P* = 2.3e^−2^) (Fig. 5e and Additional file 10: Dataset S5f). At the genome-wide level, we found weak correlation between DNA methylation and expression levels of all epigenes associated with tgDMRs (*P* = 0.2). This is because the correlation between methylation and epigene expression changes may not be straightforward, depending on the genes (housing keeping or otherwise) and location of methylation (5′ or 3′ or gene body) [11, 46, 47]. Moreover, when hyper DMRs were related to a repressor such as *ROS1*, high methylation levels can promote expression of epigenes, as observed for upregulation of expression in sensing methylation homeotsis [48] and in *Arabidopsis* allotetraploids [49]. There was a trend of elevated expression of epigenes compared to all of genes. Epigenes displayed significantly higher expression fold changes than all genes (*P* < 3.95e^−9^ in MB6S3 line and *P* < 1.28e^−8^ in BM6S3 line, Wilcoxon signed-rank test) (Additional file 1: Fig. S7h and Additional file 10: Dataset S5m). These results suggest that methylation changes in the tgDMRs may alter binding affinity of activators or repressors to alter epigene expression, as recently reported [50]. Interestingly, only three epigenes were shared between MB6S3 and BM6S3 lines, suggesting a strong effect of the nonrecurrent parents on breeding by backcross and selfing.

Notably, the loss of small RNAs in the *mop1-1* mutant has a negative effect on seedling growth and biomass heterosis, compared to the wildtype (Additional file 1: Fig. S6, b-c and Additional file 10: Dataset S5c). Moreover, seedling biomass heterosis was also decreased in the hybrid Mo17XB73:*mop1-1*. The correlation of *mop1-1* mutant with the reduced level of seedling growth was consistent with gene expression data. Among five epigenes that were associated with tgDMRs and randomly selected for quantitative RT-PCR analysis (Additional file 5: Table S4), we found that four of five (80%) epigenes tested were significantly upregulated, and the other one (*Zm00001d033278*) had the same trend of upregulation in the B73:*mop1-1* mutant (Additional file 1: Fig. S6d and Additional file 10: Dataset S5d).

Gene ontology (GO) analysis of 97 epigenes in MB6S3 and BM6S3 indicated overrepresentation of biological processes (*P* < 0.05 and fold enrichment > 2.5) in several stress responsive pathways (Fig. 5f and Additional file 10: Dataset S5g). Using RNA-seq analysis in each genotype with three biological replicates, we identified differentially expressed genes (DEGs) between the MB6S3 and B73 (3036) and between BM6S3 and Mo17 (3345). Among these DEGs, over 75% were specific to MB6S3 (2,274) or BM6S3 (2,583), and less than 24% (762) were shared in both lines (Additional file 1: Fig. S6e), which was consistent with the few overlapping DMRs (142, < 0.1%) between MB6S3 and BM6S3 (Additional file 1: Fig. S6f).

Approximately 34.5–39.5% of these tgDMRs were associated with TEs, including class I and II TEs, with no clear enrichment of specific TE families (Additional file 1: Fig. S7, a-b and Additional file 10: Dataset S5h). However, these tgDMRs were not enriched among specific TE families (Additional file 1: Fig. S7, a-b) but were associated with longer TEs (*P* < 1.2e^−4^, Wilcoxon signed-rank test) (Additional file 1: Fig. S7c and Additional file 10: Dataset S5i), LTRs with older insertion age (*P* < 1.8e^−3^, Wilcoxon signed-rank test) (Additional file 1: Fig. S7d and Additional file 10: Dataset S5j), and TEs with higher expression levels of 24-nt siRNAs (*P* < 1.4e^−3^, Wilcoxon signed-rank test) (Additional file 1: Fig. S7e and Additional file 10: Dataset S5i).

These tgDMRs were associated with TEs in their genic regions (Additional file 1: Fig. S7f and Additional file 10: Dataset S5k). Many of stress responsive genes coexist with TEs as observed in *Arabidopsis* [51] and rice [11] and may regulate expression of TE-associated genes via RdDM pathway [11]. Indeed, among eleven DEGs associated with tgDMRs in BM6S3 line, seven were involved in response to stress, including *Zm00001d026577* and *Zm00001d017987* (Additional file 1: Fig. S8, a and b). For example, *Zm00001d026577* (namely, *CP1C*) encodes one of cysteine proteases and acts as hubs in plant immunity and in abiotic stress [52]. A short DTA transposon is located in the 5′ region (− 1.5 kb from TSS) of *CP1C* (Fig. 5g). In MB6S3 line, DTA was hyper-methylated like the nonrecurrent parent Mo17, consistent with high 24-nt siRNA levels in both MB6S3 and Mo17 lines. Coincidently, *CP1C* was poorly expressed in Mo17 and nearly silenced in both MB6S3 and MB lines. The majority of 26 genes in response to various stress pathways trended to be down-regulated in two F_1_ hybrids and MB6S3 (Additional file 1: Fig. S7g and Additional file 10: Dataset S5l). This is reminiscent of a trade-off between downregulation of stress-responsive gene expression and biomass heterosis in *A. thaliana* intraspecific hybrids [53] and *Arabidopsis* allotetraploids [54].

**Fig. 6:**
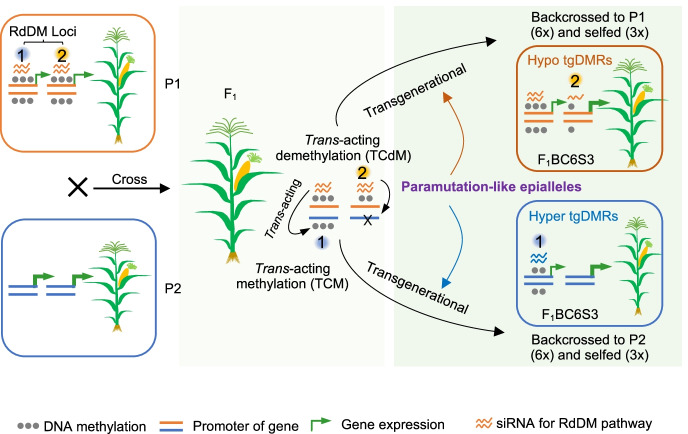
Transgenerational inheritance of hybrid-induced RdDM loci in backcrossing and selfing generations. Left panel, inbred parents (P1 and P2) have differential methylation patterns on the promoter of a gene. These loci are maintained by siRNAs via RNA-directed DNA methylation (RdDM) pathway. Hybridization triggers *trans*-acting of siRNAs to induce *trans*-chromosomal methylation (TCM) and demethylation (TCdM) loci in the F_1_ hybrid (middle panel). Note that siRNAs and gene expression are depicted only in one strand. Right panel, some of these F_1_ TCM and TCdM loci that acquired from the donor parent are heritable, like paramutation through multiple backcrossing to the recurrent parent and self-pollination due to presence of siRNAs. As a result, these transgenerational DMRs (tgDMRs) affect expression of adjacent genes (epigenes or epialleles) involved in different biological pathways including stress responses to regulate growth and adaptation in plants

These examples show a possible mechanism for siRNAs to maintain *trans*-acting methylation on a TE-associated tgDMR in the promoter to regulate expression of a stress-responsive gene. Stress-responsive genes are often associated with TEs and tend to show epigenetic regulation and inheritance in *Arabidopsis* [8, 9]. In maize, these stress-responsive genes may contribute to phenotypic variation such as seedling biomass and adaptation to environmental changes during breeding (Fig. 6). Notably, many paramutation genes are related to TE regulation [19, 55] of the genes such as *r1*, *bronze 2* (*bz2*), and *b1* loci [13, 14] involved in anthocyanin regulatory and biosynthesis pathways. These genes often activate biochemical pathways under the stress, leading to visible color phenotypes.

## Discussion

Biogenesis of 24-nt siRNAs is required for some paramutation events [19], while others involve *trans*-acting RNA transcripts [56]. In maize hybrids, the divergent population of TE-derived 24-nt siRNAs between the parents may induce RdDM in the hybrids to establish paramutation-like methylation loci, which are maintained during transgenerational inheritance (Fig. 6). These hybrid-induced DMRs are eliminated in the hybrids of *mop1-1* mutants in maize, as in the *nrpe1/nrpd1* mutant hybrids of *Arabidopsis* [29]. Thus, RdDM pathway may account for hyper DMRs induced by hybridization. For hypo DMRs, low levels of 24-nt and/or 21/22-nt siRNAs may not sufficiently maintain RdDM [42, 57], which may involve other mechanisms such as RdDM-independent pathway [58, 59], homeostasis of CHH methylation and transcriptional regulation of methylated TEs by Pol IV [41], and H3K27me3 histone modifications for non-CG methylation [60]. For example, 37% of H3K27me3-marked genes are also methylated at non-CG sites in rice [60]. They may form feedback loops, and SDG711 can catalyze H3K27me3 modification via interaction with OsDRM2 for CHH methylation.

The involvement of RdDM independent pathway may also explain why more TCdM than TCM loci tend to be transgenerational, since the amount of siRNAs may vary or dilute during selfing and backcross generations, as few siRNAs can transmit from sperm to the zygote in *Arabidopsis* [43, 61]. Among these *trans*-acting methylation loci, 5.5–6.9% hypo-DMR (TCdM) and 1.1–1.5% hyper-DMR (TCM) loci in the reciprocal hybrids can be transmitted through multiple backcross and selfing generations. This relatively low rate of transgenerational inheritance may result from the homeostasis of siRNAs in TCM loci that have initiated in the F_1_ hybrid and could be lost during subsequent RdDM process in backcross generations below the threshold levels that are required to maintain the RdDM pathway, as predicted in *A. thaliana* hybrids [62].

In maize recombination inbred lines (RILs), DNA methylation variation in one eighth-generation RIL switched from non-donor to the donor, which were guided by siRNAs [31]. However, in maize near isogenic lines, DNA methylation variation may correlate with *cis*-regulatory elements [63], independent of siRNAs. This is probably because the study using DNA methylC immunoprecipitation with microarray profiling may not effectively capture CHH methylation variation among these lines that are derived from three rounds of backcross.

While traditional paramutation events [12, 14, 64] are independent of cytoplasmic differences, paramutation-like events in mice [16], flies [17], and worms [18] are likely associated with parent-of-origin effects. In this study, B73 happened to possess a similar cytoplasm to Mo17 (Additional file 1: Fig. S8c), which may rule out a possibility of the cytoplasmic effect on establishment of these *trans*-acting epialleles in reciprocal F_1_ hybrids. However, this notion of the parent-of-origin effect on *trans*-acting epialleles remains to be investigated.

This mechanism for siRNA-mediated transgenerational inheritance is shared between plants and animals. In *C. elegans*, small RNAs contribute to transgenerational inheritance of gene expression in neurons for at least three generations via the germline Argonaute HRDE-1 [4]. Similarly, transgenerational inheritance of both gene expression and avoidance behavior to pathogens requires the Piwi Argonaute homolog and its downstream components [5]. It is notable that animals like *C. elegans* and *Drosophila* do not have obvious methylation pathways to reinforce epigenetic inheritance. In plants, the siRNA-medicated epigenetic memory is likely reinforced by the RdDM pathway. These data suggest that siRNAs can serve as epigenetic memory in both plants and animals. In plants *trans*-acting methylation induced by hybridization (internal stress) and *cis*-acting methylation induced by external stresses [65] can be transgenerational.

Notably, *trans*-acting DNA methylation changes have a strong effect from the nonrecurrent parent like paramutation on the reciprocal F_1_ hybrids. Over 779 (99.4%) tgDMRs are specific to the MB6S3 or BM6S3 line (Additional file 4: Table S3). This parent-of-origin effect is likely related to many siRNAs that are maternally transmitted during embryo and endosperm development [43, 44, 66]; these maternally derived siRNAs may exert *trans*-acting effects on establishing DNA methylation patterns in the hybrids.

## Conclusions

Transgenerational inheritance of *trans*-acting DNA methylation epialleles is reminiscent of paramutation in maize [12, 67]. Our results suggest that paramutation phenomena are more common than what has been phenotypically recorded. Transgenerational DMRs are observed in both intraspecific (this study) and interspecific [45] hybrids of maize and in the interspecific hybrids and five different allotetraploid cotton species [33]. Because many of these transgenerational DMRs are associated with the genes involved in stress response and domestication related traits [33], the hybridization-induced *trans*-acting epialleles may serve as a long-term epigenetic memory for adaptation, evolution, and domestication [15], as shown in rice [11]. As paramutation occurs in both maize and mice [13, 14], these new insights into transgenerational inheritance of *trans*-acting epigenetic changes in the plant hybrids may also shed light on epigenetic inheritance in all sexually reproducing organisms including mice and humans.

## Methods

### Plant materials

Maize F_1_ hybrids and their backcross lines were produced using two inbred lines Mo17 and B73, as illustrated in Fig. 1a. In one direction, B73 was used as the pollen donor to pollinate Mo17 and produce the F_1_ hybrid MB (Mo17xB73, by convention the maternal parent is listed first in a genetic cross). The F_1_ (MB) hybrid was backcrossed with B73 six times (MB1 to MB6), followed by self-pollination for three generations (MB6S1 to MB6S3). Likewise, the reciprocal F_1_ hybrid (BM, B73xMo17) was backcrossed with Mo17 for six generations (BM1 to BM6), followed by self-pollination for three generations (BM6S1 to BM6S3). Seeds and seedlings from these lines were used for measuring phenotypic traits, in addition to DNA and RNA analyses (see below).

To verify genetic composition, 55 plants each from MB6S3 or BM6S3 population were randomly selected for genotyping analysis using 20 pairs of Indel marker primers (Additional file 5: Table S4), with one marker on the long arm and another on the short arm of each of 10 maize chromosomes. Among them, nine BM6S3 lines and six MB6S3 lines each with the same genotype of 20 markers as the corresponding recurrent parent were further genotyped using MaizeSNP6K array [34]. Finally, BM6S3-2 (with 99.4% of the Mo17 genome) and MB6S3-3 (99.1% of the B73 genome) lines were selected to represent their respective populations for further analysis.

Two *mop1-1* (Mo17 and B73) mutant lines used in a previous study [26] were genotyped and validated by the PCR (Additional file 5: Table S4), and the homozygous mutants were used to make F_1_ hybrids and for further study.

All lines including B73, Mo17, MB, BM, MB3, MB5, BM6S1, BM6S3, BM3, BM5, MB6S1, MB6S3, B73:*mop1-1*, Mo17:*mop1-1*, and MB:*mop1-1* (Additional file 3: Table S2) were planted in a walk-in growth chamber with a light/dark cycle of 16/8 h at 28 °C/24 °C. The above ground seeding at 9 days after planting was weighed after dehydration for B73, Mo17, MB, BM, BM6S3, and MB6S3 lines each with sixteen replicates. The second leaf at 9 DAP was collected at 10 am for isolating and purifying DNA (with two biological replicates) and RNA (three biological replicates), respectively.

### Construction of MethylC-seq and DNA sequencing libraries

MethylC-library construction and sequencing were performed using with two biological replicates according to a published protocol [33]. In brief, total genomic DNA (~ 2 μg) was fragmented into 200–500 bp in size by sonication in a Bioruptor (UCD-600TS, Diagenode, Denville, NJ). The fragmented DNA was end-repaired, followed by 3-end adenylation and adapter ligation. An aliquot (~ 0.5 μg) of adapter-ligated DNA fragments was treated with bisulfateusing Zymo EZ DNA Methylation-Gold^TM^ kit (D5006), followed by 10 cycles of PCR amplification using KAPA HiFi HotStart, which was subject to purification using VAHTS DNA Clean Beads (Vazyme, N411-03). For each genotype, after quality control assessment, two (biological replicate) libraries each a unique bar code were pooled in an equal amount and sequenced across multiple lanes on an Illumina NovaSeq 6000 machine (paired-end 2 × 150 bp reads, Novogene).

For DNA sequencing library, an aliquot (~ 0.5 μg) of adapter-ligated DNA (200–500 bp) was amplified by 6 cycles of PCR using Q5 HiFi HotStart and subject to purification using VAHTS DNA Clean Beads (Vazyme, N411-03). After quality control assessment, the libraries each with a unique bar code were sequenced across multiple lanes on an Illumina NovaSeq 6000 machine (paired-end 2 × 150 bp reads, Novogene).

### Construction of mRNA-seq and small RNA-seq libraries

For each genotype, mRNA-seq libraries were constructed with three biological replicates. In brief, after DNase treatment, total RNA (1 μg) was subject to poly(A) enrichment and then fragmented into 200–500 bp in size. The fragmented mRNA was reverse-transcribed to cDNA using random hexamers, followed by 3-end adenylation and adapter ligation. cDNA was amplified by 10 cycles of PCR using Q5 HiFi HotStart and purified using VAHTS DNA Clean Beads (Vazyme, N411-03). For each genotype, mRNA-seq libraries were constructed with three biological replicates and sequenced by Illumina HiSeq X Ten system (paired-end 2 × 150 bp reads, Berrygenomics).

After DNase treatment, small RNA fraction (18–30 nt) of total RNA was recovered from a 15% urea-polyacrylamide gel. The small RNAs were ligated to 5′ and 3′ RNA oligo adapters and performed reverse transcription by amplification (10 cycles). The sRNA-seq libraries were constructed with two biological replicates for each genotype, pooled, and sequenced in one lane on an Illumina HiSeq X Ten sequencer (single-end 50 bp reads, Novogene).

### SNPs calling and genotyping

Raw paired-end sequence reads were filtered into clean data using NGSQCtookit v2.3 (http://www.nipgr.res.in/ngsqctoolkit.html). Clean reads of B73, MB6S3, and Mo17 were mapped onto the genome sequence of B73 [35] (B73_RefGen_v4, https://download.maizegdb.org/Zm-B73-REFERENCE-GRAMENE-4.0) using BWA (v0.7.15), allowing three mismatches allowed per read. Only uniquely mapped paired reads were extracted to new bam files using perl scripts, and the potential PCR duplicates were removed using “Samtools rmdup” of Samtools program (version1.3.1) [68]. The remaining reads were used for further analysis. Consecutive steps using Samtools (v0.1.19, http://samtools.sourceforge.net/) were applied for calling SNPs between B73 and Mo17 reference genomes using the pipelines described previously in rice [69]. SNPs between Mo17 and B73 were used for assessing residual Mo17 genome in MB6S3.

To analyze residual B73 genome in BM6S3, clean reads of BM6S3 and Mo17 were aligned onto the Mo17 genome sequence [36] (Mo17_CAU_V1, https://download.maizegdb.org/Zm-Mo17-REFERENCE-CAU-1.0) to call SNPs between BM6S3 and Mo17. The sequences (100 bp) flanking the B73 SNPs are extracted from Mo17_CAU_V1 and were mapped onto B73_RefGen_v4, and SNPs between BM6S3 and Mo17 in the mapped region flanking 20 bp were called for residual loci of B73 genome in BM6S3.

In another analysis, we resequenced cytoplasmic (mitochondrial and chloroplast) genomes of B73 and Mo17 accessions used in this study. Other re-sequencing data of maize and its relatives were downloaded from DDBJ (https://trace.ddbj.nig.ac.jp accession no. SRP011907) and NCBI (https://www.ncbi.nlm.nih.gov/ accession no. PRJNA528290). Chloroplast genome of B73 (accession no. AY928077.1) was downloaded from NCBI to be assembled by parsing into 200-bp bins with a step size of 20-bp. These re-sequencing data were aligned onto mitochondrial and chloroplast genomes of the reference B73_RefGen_v4 using SNP calling through the Samtools’ pipeline [68] as noted above. Heteroplasmic sequences (insertions of cytoplasmic genomes in the nuclear genome) were excluded for the analysis. Remaining SNPs from mitochondrial and chloroplast genomes were used to build neighbor-joining trees using MEGAX with 1000 replicates for bootstrap confidence analysis (Additional file 1: Fig. S8c and Additional file 10: Dataset S5n), while mitochondrial and chloroplast genomes of teosinte (*Z. may* L*.* ssp. *parviglumis*) accessions Bravo (BR) or Blanco (BL) [45] were used as outgroups.

### Mapping MethylC-seq reads

MethylC-seq reads were subject to quality control by NGSQCToolkit_v2.3 (http://www.nipgr.res.in/ngsqctoolkit.html) and then were mapped using Bismark (v0.15.0) with options (–score_min L,0,-0.2 -X 1000) [70]. Bisulphite conversion of all libraries was evaluated using the chloroplast genome (AY928077.1) as a control. The conversion rate ranged from 99.48% to 99.86%, suggesting a good reproducibility. Clean reads of 12 lines, including B73, MB3, MB5, MB6S1, MB6S3, Mo17, BM3, BM5, BM6S1, and BM6S3, were mapped onto the combine genome sequences of B73 RefGen_v4 and Mo17_CAU_V1. The ratio of uniquely mapped reads onto B73 RefGen_v4 and Mo17_CAU_V1, respectively, in each line was used to evaluate genome composition in a backcross generation.

For detecting methylation changes in backcross lines, we mapped the clean reads of B73, MB3, MB5, MB6S1, and MB6S3 onto the genome sequence of B73_RefGen_v4 [35], while the clean reads from Mo17, BM3, BM5, BM6S1, and BM6S3 were mapped onto the pseudo-Mo17 genome, from which the B73 RefGen_v4 sequences were replaced by corresponding SNPs of Mo17 [36] between Mo17 and B73. For detecting methylation changes induced by hybridization, clean reads from B73, Mo17, MB, BM, B73:*mop1-1*, Mo17:*mop1-1*, and MB:*mop1-1* lines were mapped onto the genome sequence of B73_RefGen_v4 [35]. Only the reads mapped to the unique sites covered by at least three reads were retained and used for further analysis. The reads mapped to the same sites were collapsed into a single consensus read to reduce clonal bias. To exclude a mapping bias, the overlapped cytosines among B73, Mo17, BM, and Mo17 backcross lines, including BM3, BM5, BM6S1, and BM6S3, were selected for further analysis. The same rule was applied to the overlapped cytosines among B73, Mo17, MB, and B73 backcross lines including MB3, MB5, MB6S1, and MB6S3, as well as among B73, Mo17, MB, BM, B73:*mop1-1*, Mo17:*mop1-1*, and MB:*mop1-1* lines.

### Differentially methylated regions (DMRs) of CHH context and transgenerational DMRs (tgDMRs)

CHH DMRs were identified using 200-bp sliding windows. The mean methylation level was calculated for each window [33]. For CHH DMRs, windows containing at least 32 cytosines in the CHH context covered by at least three reads were selected. DMRs between each comparison (either TCM or TCdM relative to MPV) were determined using an ANOVA test with two biological replications (*P* < 0.05) and cut-off (minimum methylation difference > 0.05).

For TCM and TCdM CHH DMRs in the F_1_ hybrids to be called as transgenerational DMRs (tgDMRs), they had to meet the two criteria using B73 backcross lines as an example. (1) They are hyper DMRs in MB6S3 compared to B73 and TCM DMRs in the MB hybrid or hypo DMRs in MB6S3 compared to B73 and TCdM DMRs in the MB hybrid. (2) For TCM DMRs in the MB hybrid, methylation level increased in all of other backcross lines including MB3, MB5, and MB6S1 compared to B73; for TCdM DMRs in the MB hybrid, methylation level decreased in all of other backcross lines including MB3, MB5, and MB6S1 compared to B73. The same criteria were applied to the identification of tgDMRs in Mo17 backcross lines.

### Analysis of small RNA-seq data

For small RNA-seq data analysis each with two biological replicates, the raw reads were trimmed from adaptors to retain 18- to 30-nt reads using Cutadapt (v 1.9.1, https://cutadapt.readthedocs.org/en/stable/) and parsed using the NGSQCToolkit_v2.3 (http://www.nipgr.res.in/ngsqctoolkit.html) for quality control. Only 18–30-nt long reads were retained and mapped onto B73 RefGen_v4 [35] using Bowtie (v1.1.0) [71], allowing only one unique hit (-m 1) and zero mismatch. After removing the structural RNAs, including rRNA, tRNA, snoRNA, and snRNA fragments, the mapped reads were normalized as reads per ten million (RPTM) [69].

### Analysis of mRNA-seq data each with three biological replicates

As a quality control, all reads were parsed using NGSQCToolkit_v2.3 (http://www.nipgr.res.in/ngsqctoolkit.html). The cleaned RNA-seq reads from 12 libraries (four genotypes including B73, BM, MB, and MB6S3) were mapped onto B73 RefGen_v4 [35]. Cleaned reads from six libraries (Mo17 and BM6S3 lines) were mapped onto the pseudo-Mo17 genome. TopHat soft (v2.1.1) was used for mapping the reads [72]. Uniquely mapped reads were extracted by Perl scripts to determine transcript values by Cufflinks (v2.2.1) [73]. The transcript levels were quantified by FPKM as previously described [69]. Normalized expression values (FPKM) on a log2 scale were used to evaluate sample relationships by principal component analysis (PCA). Differential expression analysis was performed using edgeR [74], and the differentially expressed genes (DEGs) were identified using both the fold-change (> 1.5) and analysis of edgeR (FDR< 0.05). Gene Ontology (GO) enrichment analysis was performed using the agriGOv2 Tool (http://systemsbiology.cau.edu.cn/agriGOv2/).

### Analysis of transgenerational DMRs and small RNAs between W22 and Teosinte

The WGBS data, WGS data, and sRNA-seq data published by [45] were downloaded from the National Center for Biotechnology Information (NCBI) with the BioProject accession numbers PRJNA526266, PRJNA528290 and PRJNA528352. The analysis used the reference genome of W22 (https://download.maizegdb.org/Zm-W22-REFERENCE-NRGENE-2.0) [38] and the bioinformatics’ pipelines outlined above.

### Analysis of structural variation (SV) and full-length LTRs

To identify SV, Mo17 genome were divided into 10-kb windows with 100-bp steps (100x depth of genome) and then mapped onto B73 genome (RefGen_v4) using minimap2 software (v2.18-r1015) with default parameters [75]. Mapped results were sorted by Samtools to call SV using cuteSV (v1.0.11) with options “-s 10 –r 500 -l 50 -sl 50” [76]. A total of 175,849 SV were identified, including 94,779 DEL (deletion, 107,516,448 bp of B73-specific sequences, 59.8%), 77,705 INS (insertion, 62,043,121 bp of Mo17-specific sequences, 34.5%), 2784 DUP (duplications), 535 INV (inversion), and 46 TRL (translocation). Approximately 2.1% (7/327) tgDMRs in the Mo17 backcross lines and 3.9% (18/462) tgDMRs in the B73 backcross lines overlapped with breakpoints of SV, which was higher than 1.3% (58,721/4,645,542) in whole genome.

To investigate insertion age of long-termianl repeats (LTRs), the full-length LTRs of B73 genome (v4) was identified using LTR_FINDER (v1.0.6) [77]. The age was estimated using the mutation rate of 6.5 × 10^−9^ in graases [78].

### Analysis of other epigenetic data

Published epigenetic datasets were downloaded from NCBI (accession no. PRJNA381532, PRJNA527732), including Dnase-seq [39], ATAC-seq, and ChIP-seq of histone modification (H3K27me3, H3K36me3, H3K56ac, H3K4me3, H3K4me1) [40] to investigate chromatin state of tgDMRs. All raw reads were parsed using NGSQCToolkit_v2.3 (http://www.nipgr.res.in/ngsqctoolkit.html), and then clean reads were mapped onto B73 RefGen_v4 [35] using BWA (v0.7.15). Mapped reads with mapping quality of ≥ 10 were extracted to new bam files, and the potential PCR duplicates were removed using Picard-tools (version 2.0.1) (http://broadinstitute.github.io/picard/). The remaining reads were normalized as reads per million (RPM) for furthe analysis with statistical tests.

### Quantitative RT–PCR assays

To determine the effect of siRNAs on tgDMRs and gene expression, five epigenes each associated with one or more tgDMRs in the B73 backcross lines were randomly selected for analysis. HiScript® II Reverse Transcriptase (RNase H-) (Vazyme, R223-01, Vazyme Biotech Co., Ltd., Nanjing, China) was used to transcribe mRNA into cDNA for qPCR assays using a primer pair for each gene (Additional file 5: Table S4) in the reaction of AceQ® qPCR SYBR Green Master Mix (SYBR Green I) (Vazyme, Q111-02).

## Supplementary Information

**Additional file 1: Figure S1**. Genotyping results and DNA methylation changes in F_1_ hybrids and backcross lines. **Figure S2**. Hybrid-induced methylation changes were conserved in two F_1_ hybrids and in all backcross lines. **Figure S3**. Transgenerational DMRs resemble the methylation levels of the donor parent. **Figure S4**. Genomic features of tgDMRs. **Fig. S5**. Roles of siRNAs in the initiation and maintenance of TCM and TCdM loci. **Figure S6**. Correlation between transgenerational DMR and gene expression levels. **Figure S7**. Characteristics of the TEs associated with tgDMRs. **Figure S8**. Transgenerational hypermethylated elements targeted by siRNAs in stress-responsive genes.

**Additional file 2: Table S1**. Nine BM6S3 and six MB6S3 lines were genotyped using MaizeSNP6K.

**Additional file 3: Table S2**. Summary of high-throughput data for backcrossing lineages.

**Additional file 4: Table S3**. Transgenerational hybrid-induced DMRs for backcross lines.

**Additional file 5: Table S4**. Primer pairs used in this study.

**Additional file 6: Dataset S1**. Hybrid-induced DNA methylation changes are transgenerational in multiple generations of backcrossing and selfing.

**Additional file 7: Dataset S2**. Transgenerational DNA methylation states resemble the nonrecurrent parent.

**Additional file 8: Dataset S3**. Initiation and transmission of transgenerational TCM and TCdM loci dependent on siRNAs.

**Additional file 9: Dataset S4**. Transgenerational DNA methylation in backcross lines derived from modern maize W22 and teosinte.

**Additional file 10: Dataset S5**. Transgenerational methylation loci correlate with gene expression levels.

**Additional file 11.** Review history.

## Data Availability

All sequencing data are available under NCBI under the BioProject accession number PRJNA638210 (https://www.ncbi.nlm.nih.gov/bioproject/PRJNA638210) [79]. All datasets generated and/or analyzed in study are available in the article, the source data files that accompany Figs. 1, 2, 3, 4, 5 and 6 and Figs. S1-8, Tables S1, S2, S3 and S4, and Datasets S1, S2, S3, S4 and S5. Published epigenetic datasets used in this study included Dnase-seq [39], ATAC-seq, and ChIP-seq of histone modifications (H3K27me3, H3K36me3, H3K56ac, H3K4me3, H3K4me1) [40] (NCBI accession no. PRJNA381532, PRJNA527732), and WGBS data, WGS data, and sRNA-seq data [45] (NCBI accession numbers PRJNA526266, PRJNA528290, and PRJNA528352).
