# Supplementary material for: Small RNAs mediate transgenerational inheritance of genome-wide trans-acting epialleles in maize
Source: Genome Biol. 2022 Feb 9;23:53. doi: 10.1186/s13059-022-02614-0 (PMC8827192; doi:10.1186/s13059-022-02614-0)
Supplement: Supplementary file 1 — Additional file 1: Figure S1. Genotyping results and DNA methylation changes in F1 hybrids and backcross lines. Figure S2. Hybrid-induced methylation changes were conserved in two F1 hybrids and in all backcross lines. Figure S3. Transgenerational DMRs resemble the methylation levels of the donor parent. Figure S4. Genomic features of tgDMRs. Fig. S5. Roles of siRNAs in the initiation and maintenance of TCM and TCdM loci. Figure S6. Correlation between transgenerational DMR and gene expression levels. Figure S7. Characteristics of the TEs associated with tgDMRs. Figure S8. Transgenerational hypermethylated elements targeted by siRNAs in stress-responsive genes. [file 13059_2022_2614_MOESM1_ESM.pdf]

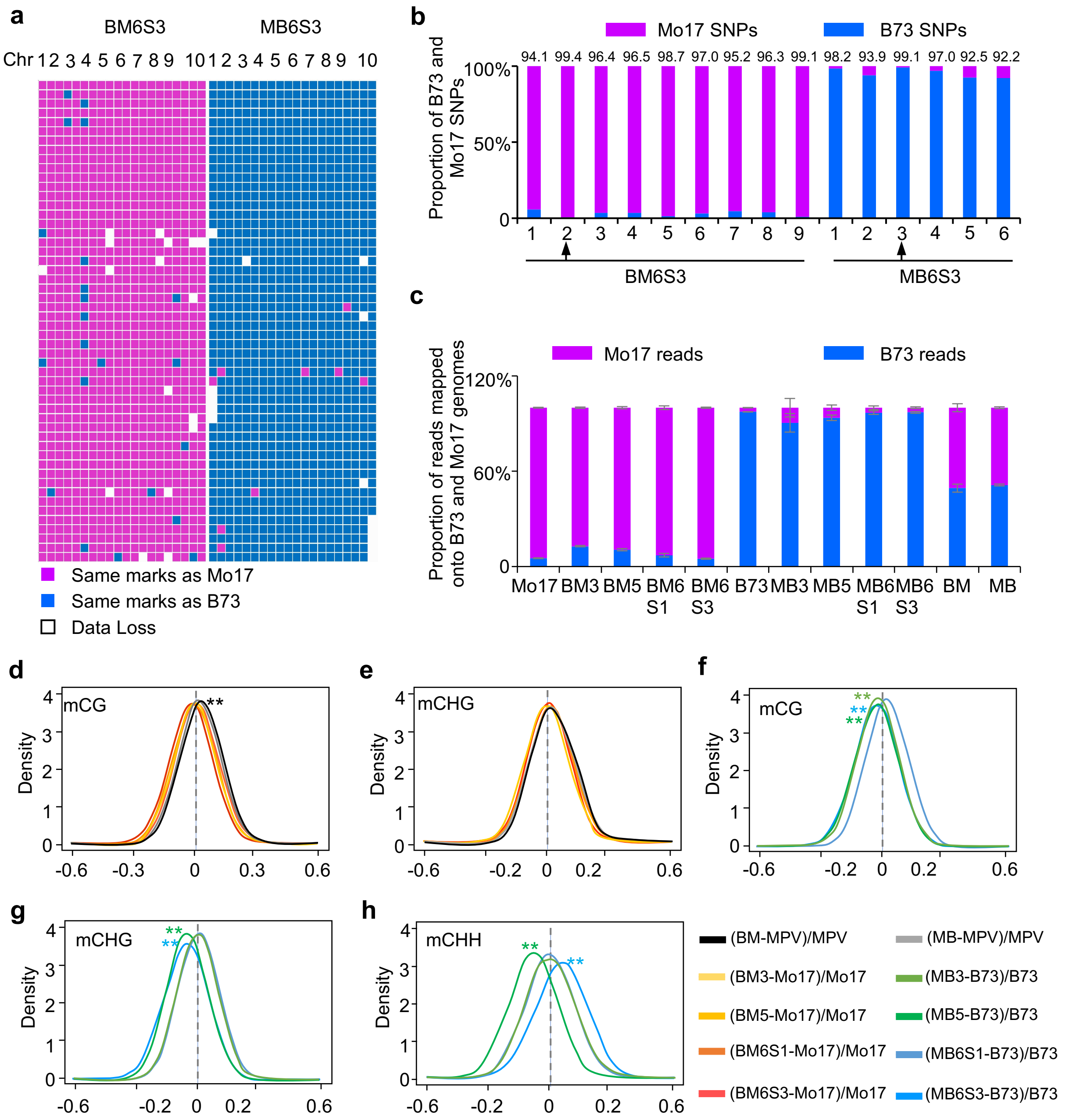

Additional file 1: Fig. S1 . Genotyping results and DNA methylation changes in F1 hybrids and backcross lines. (**a**) Genomic conversion of BM6S3 and MB6S3 populations to the recurrent parent. A set of 55 randomly selected plants each was genotyped using 2 Indel markers per chromosome (one from short and another from long arm). Chromosome 1-10 are shown above the grid with markers from Mo17 (orange), B73 (blue), and data loss (white). (**b**) Proportion of SNP frequency in nine BM6S3 and six MB6S3 lines that were genotyped using MaizeSNP6K with Mo17 (violet) and B73 (navy) SNPs, respectively. Two lines (arrow) were used for resequencing. (**c**) Proportion (mean±SD) of sequence reads that were uniquely mapped onto the Mo17 or B73 genome. Reads from all MethylC-seq libraries of 12 maize lines were used to map onto the combined genome sequence of B73 and Mo17 with Mo17 (violet) and B73 (navy) reads, respectively. (**d**, **e**) Kernel density (*y*-axis) distribution of CG (**d**) and CHG (**e**) methylation level changes (*x*-axis) in F_1 hybrids compared to the mid-parent value (MPV) and in the backcross lines relative to their recurrent parents. Density is estimated by the difference (I-II) divided by II (with a 100-kb bin window). Line colors indicate BM (black), MB (gray), BM3 (light yellow), BM5 (yellow), BM6S1 (orange), and BM6S3 (red), respectively. Double asterisks indicate the statistical significance level of $P < 0.01$ (two-sided Wilcoxon signed-rank test). (**f-h**) Kernel density (*y*-axis) distribution of CG (**f**), CHG (**g**) and CHH (**h**) methylation level changes (*x*-axis) in the backcross lines compared to their recurrent parents, as estimated in (**d**) and (**e**). Line colors indicate MB3 (light green), MB5 (green), MB6S1 (light blue), and MB6S3 (blue), respectively. Double asterisks indicate statistical significance ($P < 0.01$, two-sided Wilcoxon signed-rank test).

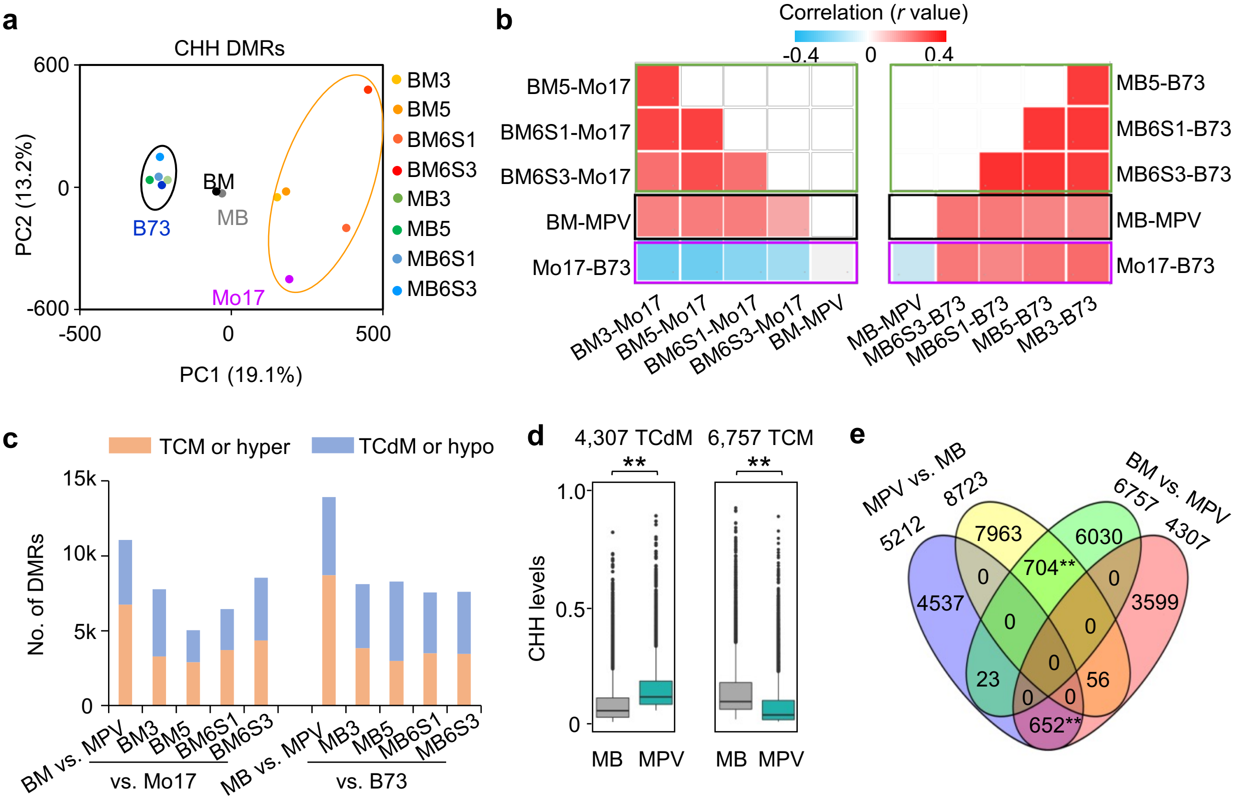

Additional file 1: Fig. S2 . Hybrid-induced methylation changes were conserved in two F_1 hybrids and in all backcross lines.

(**a**) Principal component analysis (PCA) for CHH methylation levels of all genotypes with the overall variance explained by PC1 (*x*-axis) and PC2 (*y*-axis). Circles indicate the clustered samples derived from backcrossing to B73 (navy) and to Mo17 (violet), respectively, with two F_1 hybrids in the middle.

(**b**) Pairwise comparison of correlation coefficients of the methylation changes in the two F_1 hybrids relative to MPV and in the backcross lines relative to their recurrent parents, with high correlation between backcross lines compared to their recurrent parents (green box), followed by the two F_1 hybrids and backcross lines (black box), and between Mo17 and B73 and backcross lines (purple box). The Pearson's product-moment correlation coefficients are shown in the scale of *r* values.

(**c**) Number of hyper DMRs (TCM, light orange) and hypo DMRs (TCdM, light blue) in the F_1 hybrids relative to MPV and in the backcross lines relative to their recurrent parents.

(**d**) CHH methylation levels (*y*-axis) of the TCdM (left) and TCM (right) in MB relative to MPV. A double asterisk indicates the statistical significance level of $P < 0.05$ (two-tailed Student's *t*-test).

(**e**) Venn-diagram analysis for the number of DMRs in BM and MB hybrids. Double asterisks indicate statistical significance ($P < 0.01$, Hypergeometric test).

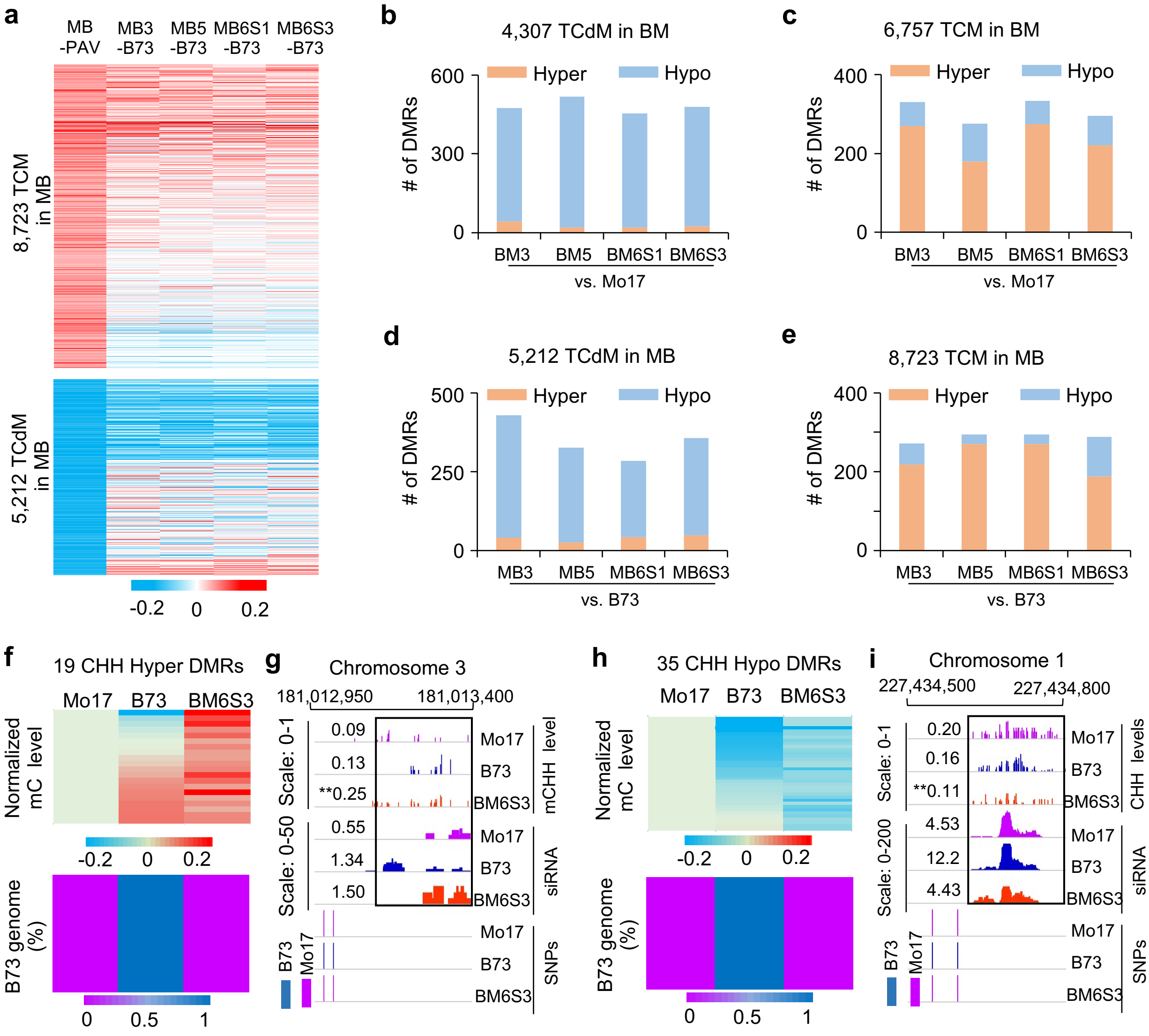

Additional file 1: Fig. S3 . Transgenerational DMRs resemble the methylation levels of the donor parent.
(**a**) Heatmaps show increased methylation levels in 8,723 TCM loci (upper panel) and decreased methylation levels in 5,212 TCdM loci (lower panel) in all backcrossing and selfing lines examined.
(**b**, **c**) Some of TCM and TCdM loci in BM hybrid lines were inherited to BM3, BM5 , BM6S1, and BM6S3, respectively.
(**d**, **e**) Some of TCM and TCdM loci in MB hybrid lines were transmitted to MB3 , MB5 , MB6S1 and MB6S3, respectively.
(**f**, **h**) Transgenerational inheritance of 19 hyper DMRs (**f**) and 35 hypo DMRs (**h**) in BM6S3 lines (upper panels). Genomic regions in corresponding DMRs were converted to the recurrent parent Mo17 (lower panels). The percentage was estimated by the average B73 allele ratio in each DMR from all loci with SNPs.
(**g**, **i**) Examples of Chr3: 181013201-181013400 (**g**) showing high methylation and siRNA levels in both BM6S3 and B73, and of Chr1: 227434601-227434800 (**i**) displaying low methylation and siRNA levels in both of BM6S3 and B73. The numbers indicate average levels of DMR methylation and siRNA expression (RPTM). Double asterisks indicate statistically significant methylation changes between BM6S3 and its nonrecurrent parent Mo17 ($P < 0.05$, one-way ANOVA test for methylation and two-tailed Student's t-test for siRNA expression). The scales were 0-1 and 0-50 for normalized methylation and siRNA levels, respectively.

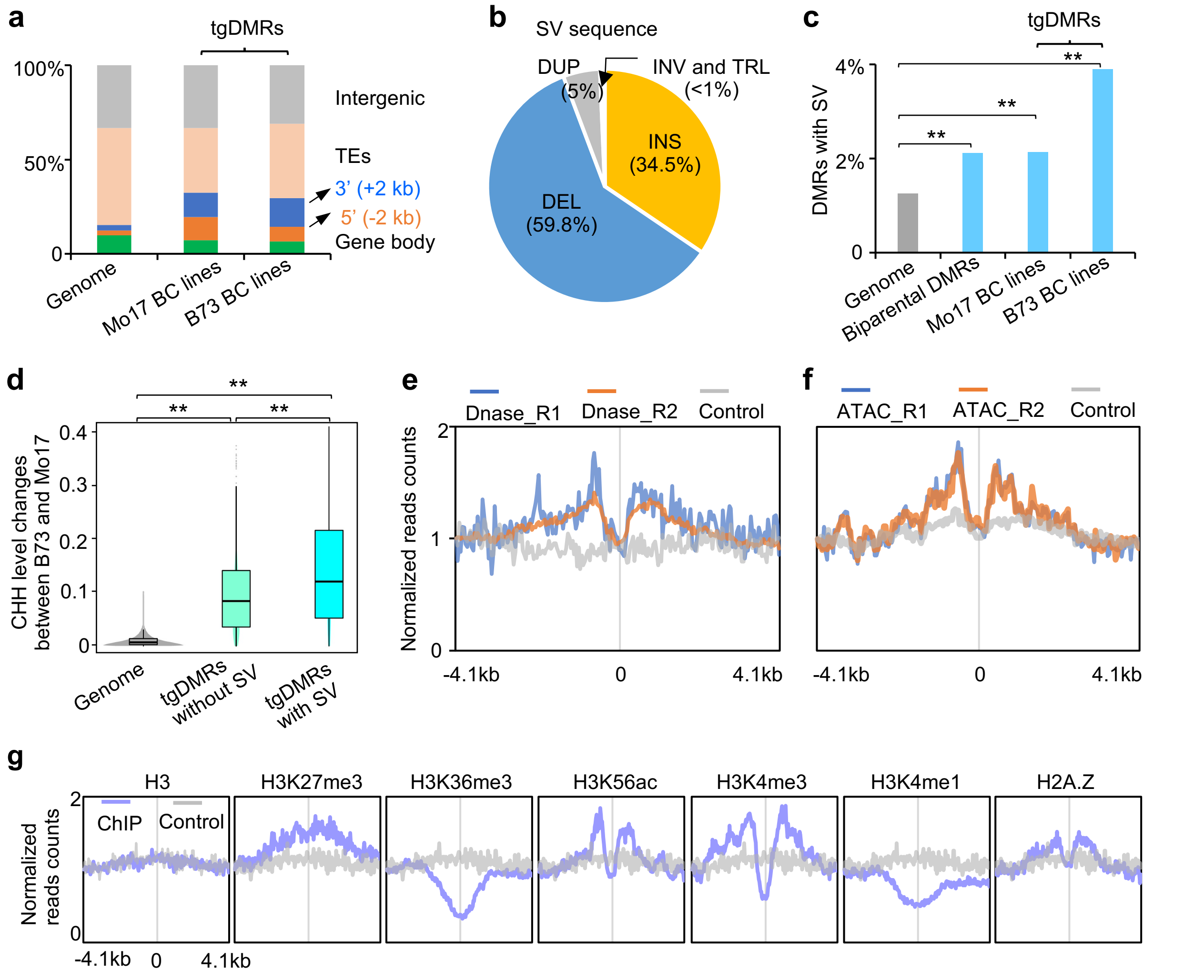

Additional file 1: Fig. S4. Genomic features of tgDMRs.

(**a**) Fraction of tgDMRs in different genomic features, including gene body, 5' (-2 kb to the transcription start site, TSS) and 3' (transcription termination site, TTS, to +2 kb) regions, TEs, and intergenic regions (excluding TEs) in Mo17 and B73 backcross lines, and whole genome (genome).

(**b**) Pie chart showing the relative composition (%) of total sequence of structural variation (SV) between B73 and Mo17 in different categories. DEL: deletion; DUP: duplication; INS and TRL: insertion and translocation; INS: insertion.

(**c**) Percentage of DMRs with SV (*y*-axis) in bipatental, Mo17 BC line, and B73 BC line tgDMRs (*x*-axis). Double asterisks indicate statistical significance ($P < 0.01$, Chi-square test).

(**d**) CHH methylation levels (*y*-axis) of tgDMRs without SV, tgDMRs with SV, and whole genome (genome). Double asterisks indicate statistical significance ($P < 0.01$, wo-sided Wilcoxon signed-rank test).

(**e-f**) Normalized read counts (reads per million, RPM, *y*-axis) of Dnase-seq (**e**) and ATAC-seq (**f**) in the regions flanking the tgDMRs. Location of read loci (*x*-axis) is centered (0) relative to tgDMRs. Colors indicate replicate 1 (R1, blue), replicate 2 (R2, orange), and an equal mix of random regions as the genome-wide control (grey).

(**g**) Normalized read counts (RPM, *y*-axis) of histone modification data, centered on tgDMRs. *x*-axis indicates location of read loci relative to the center (0) of tgDMRs. Colors indicate ChIP-seq (blue) data and an equal mix of random regions (control, grey).

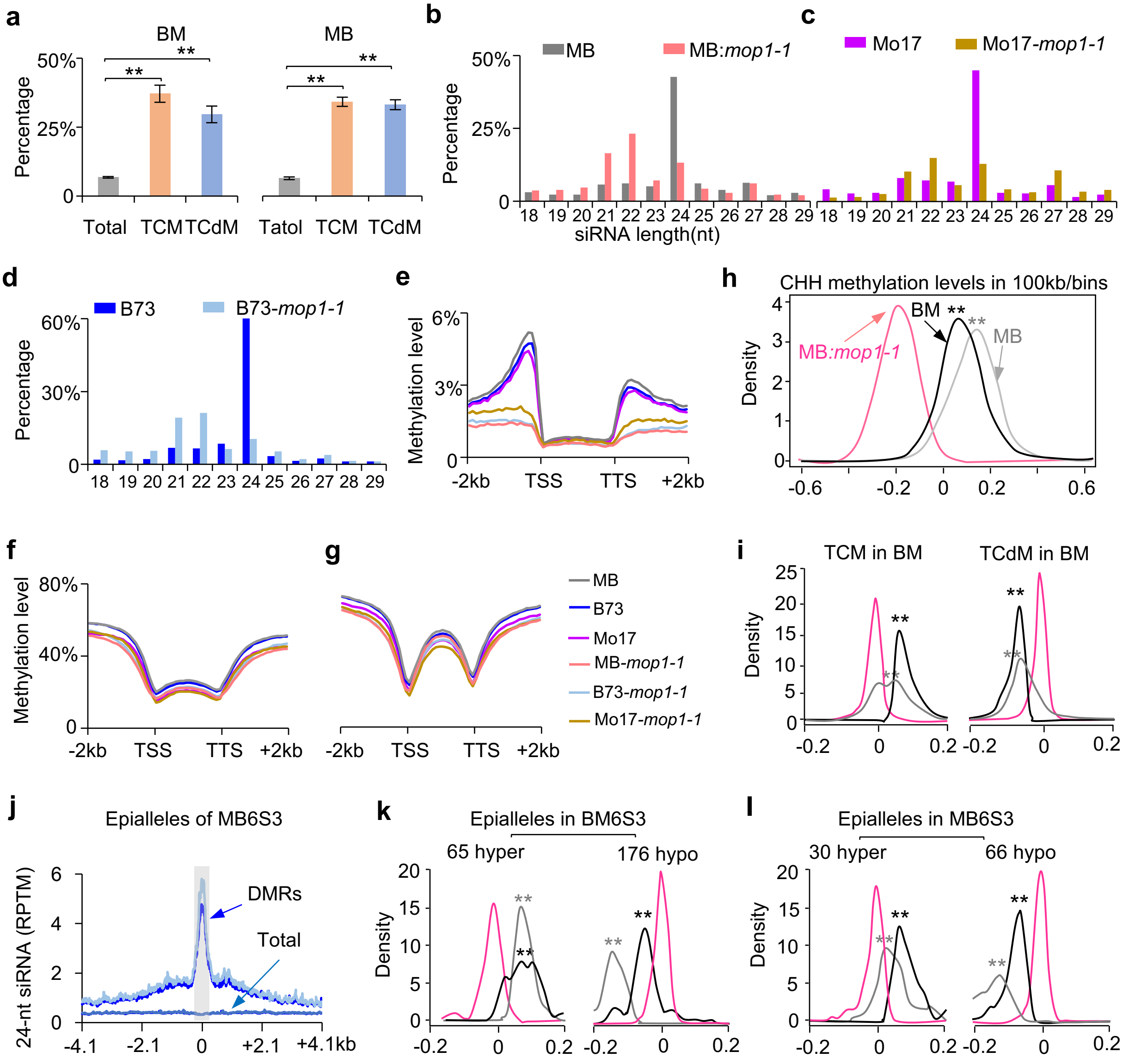

Additional file 1: Fig. S5. Roles of siRNAs in the initiation and maintenance of TCM and TCdM loci.
(**a**) The expression of 24-nt siRNA (mean±SD) enriches on the TCM and TCdM loci of the BM (left panel) and MB (right panel) lines. Double asterisks indicate the statistical significance level of $P<0.01$ (two-tailed Student's *t*-test). SMRs: similar methylation regions between the hybrid and MPV.
(**b-d**) Loss of 24-nt siRNAs (*y*-axis) in the MB-*mop1-1* hybrid (**b**), B73-*mop1-1* (**c**), and Mo17-*mop1-1* (**d**) lines. Colors indicate MB (grey), B73 (navy), Mo17 (violet), MB-*mop1-1* (pink), B73-*mop1-1* (light blue), and Mo17-*mop1-1* (gold).
(**e-g**) Decreased methylation levels (*y*-axis) of CHH (**e**), CHG (**f**) and CG (**g**) context in B73-*mop1-1*, Mo17-*mop1-1* and MB-*mop1-1* lines compared with their corresponding wild type in the genic region (*x*-axis). Line colors indicate MB (grey), B73 (navy), Mo17 (violet), MB-*mop1-1* (pink), B73-*mop1-1* (light blue), and Mo17-*mop1-1* (gold).
(**h**) Kernel density (*y*-axis) distribution of CHH methylation level changes (*x*-axis) in F_1 hybrids compared to their respective MPV. Line colors indicate MB (grey), MB-*mop1-1* (pink), BM (black). Double asterisks indicate statistical significance ($P < 0.01$, two-sided Wilcoxon signed-rank test).
(**i**) Density distribution of methylation levels (*y*-axis) in the BM (black), MB (gray), and MB-*mop1-1* (pink) hybrid lines relative to their respective MPV using TCM (left panel) and TCdM (right panel) loci. *x*-axis: scale of changes (0 = no change) in the F_1 hybrid. Double asterisks indicate statistical significance ($P < 0.01$, two-sided Wilcoxon signed-rank test).
(**j**) Levels of 24-nt siRNAs (RPTM, *y*-axis) in MB6S3 (light blue) and its recurrent parent B73 (navy). *x*-axis indicates location of siRNA loci relative to the center (light grey) of epialleles in the MB6S3 line. "Total" represents siRNA expression from all windows of the whole genome.
(**k**, **l**) Density distribution of methylation levels (*y*-axis) in the BM (black), MB (gray), and MB:*mop1-1* (pink) hybrids relative to MPV, as estimated in (**i**) using 65 hyper epialleles (**k**, left panel) and 176 hypo epialleles (**k**, right panel) in the BM6S3 line and 30 hyper epialleles (**l**, left panel) and 66 hypo epialleles (**l**, right panel) in the MB6S3 line. Double asterisks indicate statistical significance ($P < 0.01$, (two-sided Wilcoxon signed-rank test).

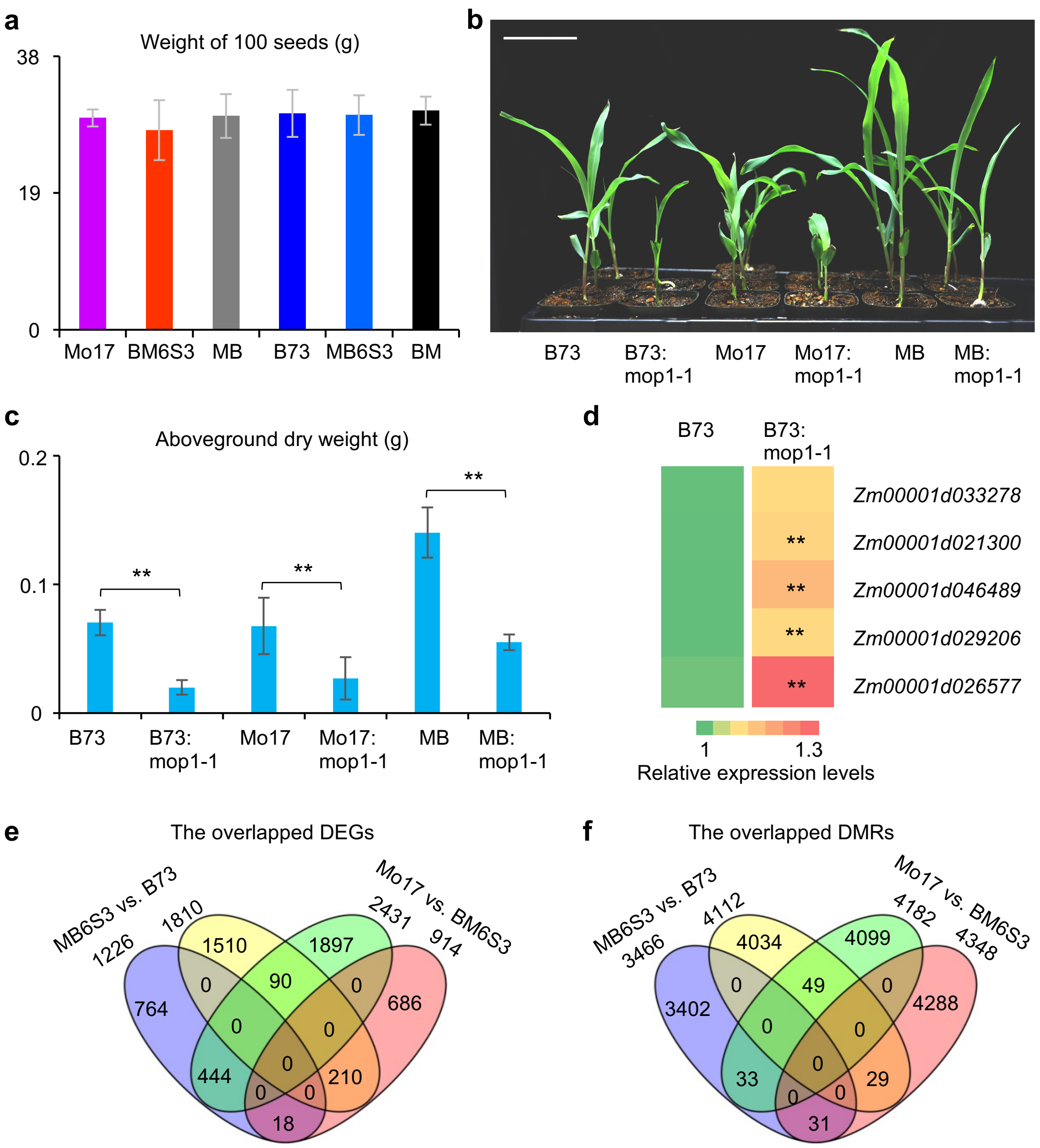

Additional file 1: Fig. S6. Correlation between transgenerational DMR and gene expression levels.
(**a**) Weight of 100 seeds (means ± SD) in Mo17, BM6S3, MB, B73, MB6S3 and BM.
(**b, c**) Seedling phenotypes (**b**) and aboveground dry weight (means ± SD) (**c**) at the same stage in B73 (n = 3) and B73:*mop1-1* (n = 2) (left), Mo17 (n = 4) and Mo17:*mop1-1* (n = 3) (middle), and MB (n = 3) and MB:mop1-1 hybrids (n = 3) (right). Photos were taken at the same time with the same scale (bar = 10 mm). Double asterisks indicate statistical significance ($P < 0.05$, two-tailed Student's *t*-test).
(**d**) Relative expression levels (R.E.L) of five epigenes by qRT-PCR analysis. *18s rRNA* (*AF168884*) was used as an internal control. Double asterisks indicate the statistical significance ($P < 0.05$, two-tailed Student's *t*-test).
(**e**, **f**) Numbers of the overlapped DEGs (**e**) and DMRs (**f**) between backcross lines MB6S3 and BM6S3, compared to their corresponding recurrent parents B73 and Mo17, respectively. The numbers indicate upregulated genes or hyper DMRs from each comparison.

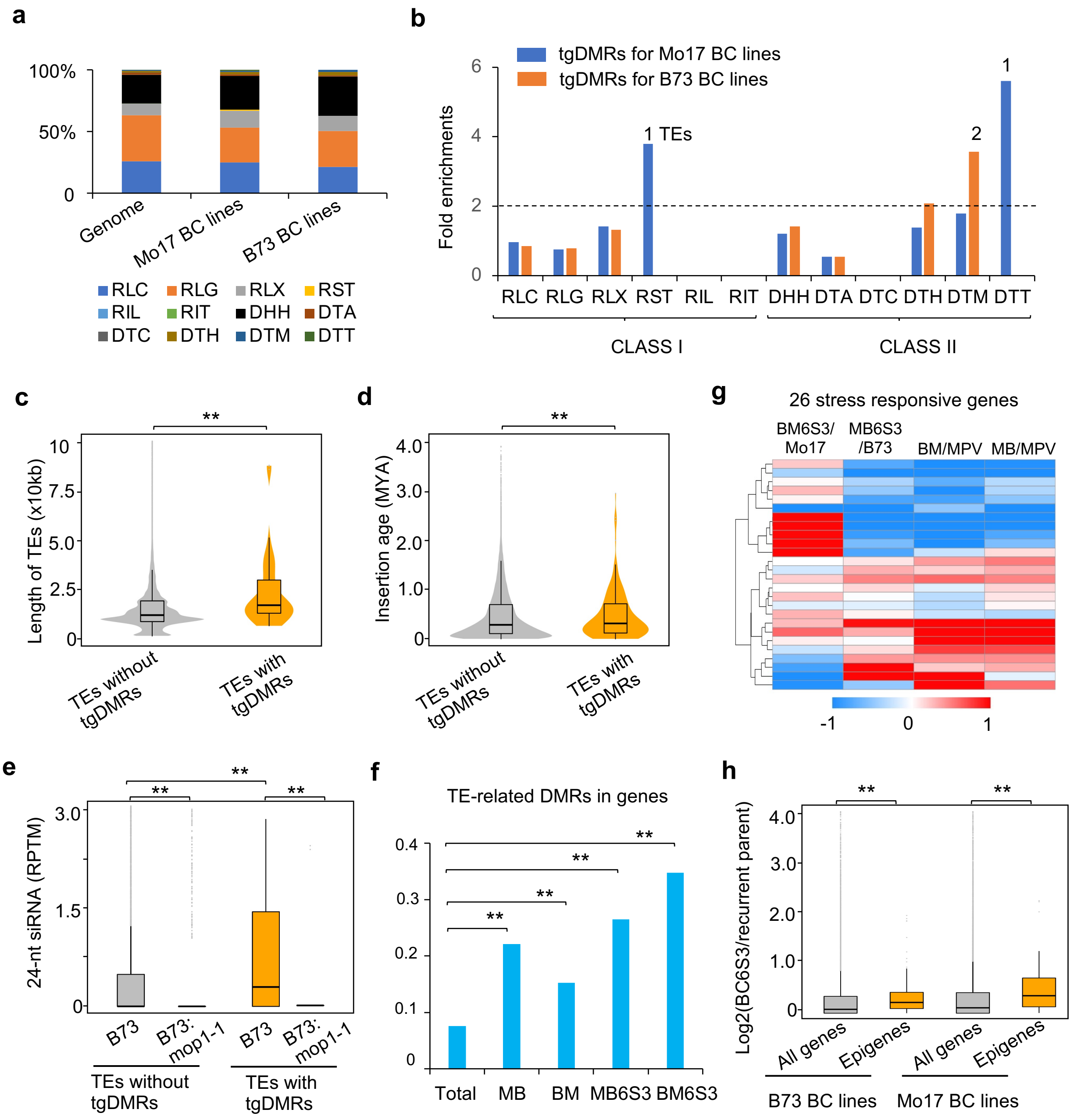

Additional file 1: Fig. S7. Characteristics of the TEs associated with tgDMRs.

(**a**) Fraction of tgDMRs in different TEs families, including class I (RLC, RLG, RLX, RST, RIL, RIT) and class II (DHH, DTA, DTC, DTH, DTM, DTT).

(**b**) Enrichment of TE families in tgDMRs. The dotted line indicates statistic significance ($P < 1e^{-5}$, Fisher'sexact test).

(**c**) Length (*y-axis*) of TEs in tgDMRs. Double asterisks indicate statistical significance level ($P < 0.01$, two-sided Wilcoxon signed-rank test).

(**d**) Insertion age (*y-axis*) of LTRs in tgDMRs. Double asterisks indicate statistical significance level ($P < 0.01$, two-sided Wilcoxon signed-rank test).

(**e**) Expression levels (*y*-axis) of 24-nt siRNAs from TEs in tgDMRs. Double asterisks indicate statistical significance ($P < 0.01$, two-sided Wilcoxon signed-rank test).

(**f**) Percentage (*y*-axis) of TEs-related DMRs in genes. *x*-axis: TCM and TCdM in MB, TCM and TCdM in BM, tg-epialleles in MB6S3, tg-epialleles in BM6S3. Double asterisks indicate statistical significance ($P < 0.01$, Hypergeometric test).

(**g**) Heatmaps show expression level changes of 26 epiallele-associated stress-responsive genes in BM6S3 compared to Mo17, MB6S3 compared to B73, and two F_1 hybrids compared to MPV.

(**h**) Absolute value (*y*-axis) of log_2 expression ratios (BC6S3/recurrent parent) in all genes and epigenes. BC6S3 indicate last backcross generations (MB6S3 and BM6S3). Double asterisks indicate statistical significance ($P < 0.01$, two-sided Wilcoxon signed-rank test).

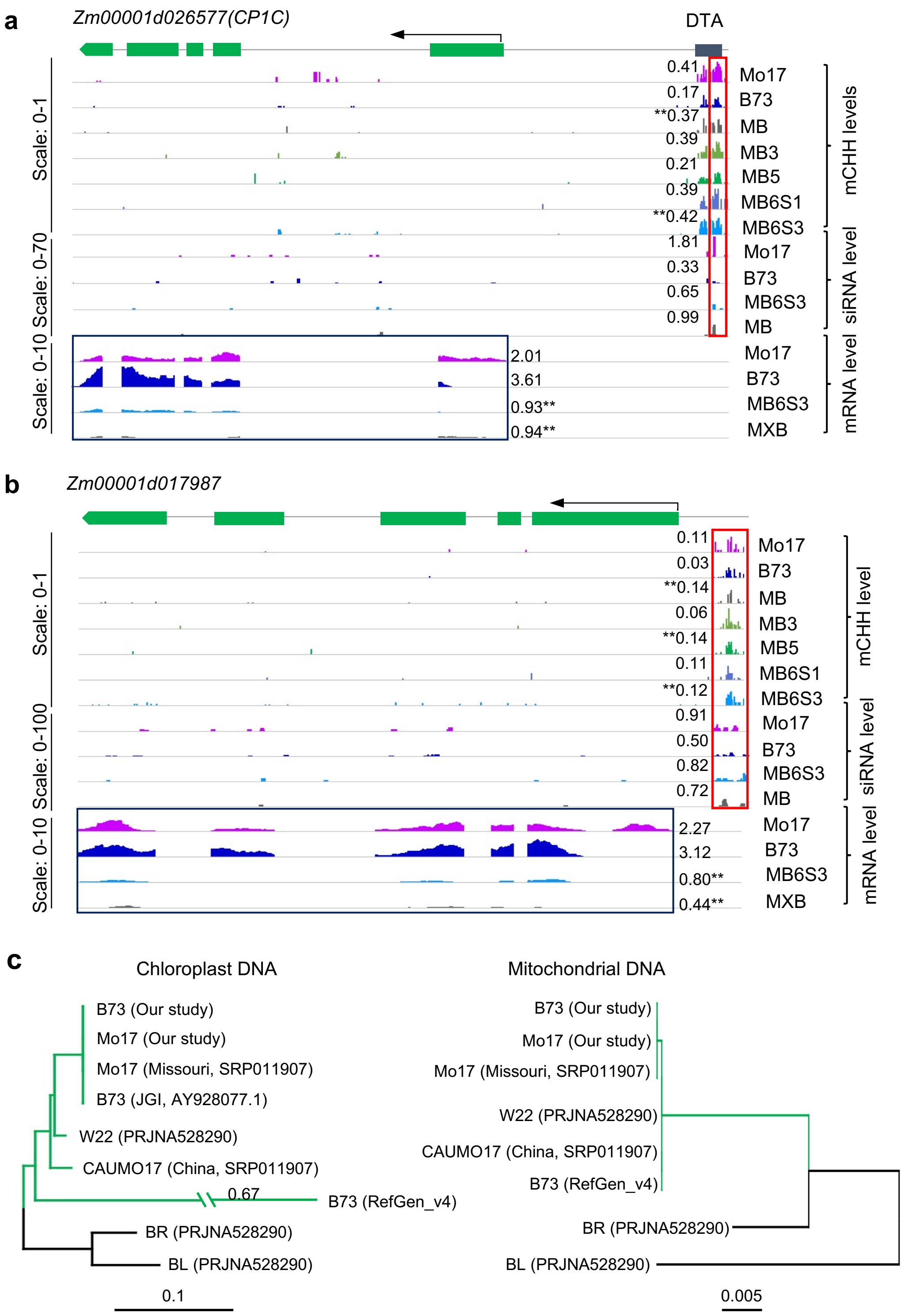

Additional file 1: Fig S8. Transgenerational hypermethylated elements targeted by siRNAs in stress-responsive genes.

(**a**, **b**) Examples of transgenerational hypermethylated promoter regions correlated with reduced expression levels of *Zm00001d026577* (**a**) and *Zm00001d017987* (**b**). Boxed regions show changes in transgenerational hyper methylation and siRNA expression (red) and in gene expression levels (black). Each locus was depicted with exons (green) and transcriptional direction (arrow). Numbers indicate average levels of DMR methylation, siRNA (RPTM), and gene (FPKM) expression. Double asterisks indicate statistically significant changes between MB hybrid and MPV or between a backcross line and its recurrent parent ($P < 0.05$, one-way ANOVA test for methylation and two-tailed Student's *t*-test for siRNA and gene expression).

(**c**) Phylogenetic tree was reconstructed by the Neighbor-joining (NJ) using SNPs of chloroplast genome (left panel) and mitochondrial genome (right panel). The scale indicates branch length. Chloroplast and mitochondrial genomes of teosinte (*Z. may* L. ssp. *parviglumis*) accessions Bravo (BR) or Blanco (BL) [45] were used as outgroups.
