# Supplementary material for: Small RNAs mediate transgenerational inheritance of genome-wide trans-acting epialleles in maize
Source: Genome Biol. 2022 Feb 9;23:53. doi: 10.1186/s13059-022-02614-0 (PMC8827192; doi:10.1186/s13059-022-02614-0)
Supplement: Supplementary file 11 — Additional file 11. Review history. [file 13059_2022_2614_MOESM11_ESM.docx]

Review History

**First round of review**

**Reviewer 1**

**Are you able to assess all statistics in the manuscript, including the appropriateness of statistical tests used?**

Yes, and I have assessed the statistics in my report.

**Comments to author:**

The manuscript titled "Small RNAs mediate transgenerational inheritance of genome-wide trans-acting epialleles in maize" by Chen et al. characterized the effect of hybridization on the DNA methylome in maize and followed the DNA methylome in backcrossing and selfing progenies. They identified hundreds of genomic loci that exhibited inheritance of hybrid-induced epigenetic changes (called tgDMRs). They also showed the correlation between sRNA levels and DNA methylation levels at these loci through small RNA sequencing. Importantly, mop1 hybrids were used to demonstrate that sRNA is necessary for hybrid-induced DNA methylation changes. In the end they examined the correlation between DNA methylation, sRNA and gene expression of selected genes. Transgenerational inheritance is clearly an important topic in epigenetics, and I think the experiment design is fine to answer the question(s) proposed. The overall logic of the manuscript is relatively easy to follow but main concerns exist, particularly on the presented DNA methylome data.

1. A clear definition of tgDMR is needed in the main text. I think the intuitive (and most strict) definition for tgDMRs are the ones that were identified as DMRs in all F1 and offspring generations. Considering the metastable nature of CHH methylation and measurement errors, this criterion can be slightly relaxed. However, I feel that multiple definitions of tgDMR were mixed in the text. A main table listing the number of DMRs should be included.

a. Page 8, "a fraction of hybrid induced hyper- or hypo-DMR loci persisted over nine generations of backcrossing followed by selfing ……. These hyper (TCM) and hypo (TCdM) DMRs are called transgenerational DMRs (tgDMRs)". After reading the information from "Methods details", I'm still not clear whether these DMRs are "normal" DMRs (all contexts included) or CHH DMRs, or whether both criteria are satisfied as indciated in page 24.

b. Assuming the 2 (both) criteria in page 24 were used for defining tgDMRs, numbers are inconsistent here and there in the main text.

Page 8: 327 and 462 tgDMRs in Mo17 and B73 backcross lines;

Page 9: 19 hyper tgDMRs and 60 hypo DMRs in MB6S3 population;

Page 9, paragraph 2: 19 hyper tgDMRs and 35 hypo tgDMRs in BM6S3 lines (whereas Figure S3 heatmaps indicates many more DMRs);

Page 9, last paragraph: 36.1% (118/327) and 31.0% (143/462) … were stably transmitted to the offspring through six backcrossing and three selfing generations (weren't all the tgDMRs stably transmitted? 118 and 143 are larger than 19+ 60 or 19 + 35?).

Page 8, paragraph 2: 7-10% TCdM DMRs in the BM hybrid were also hypo tgDMRs …, and 2-3% TCM DMRs in the BM hybrid also remained hyper tgDMRs … (it seems that tgDMRs here were used to indicate the same direction of methylation changes, instead of solely referring to the BM/MB6S3 generation).

Page 8: "threefold or more hypo DMRs (7-10% for Mo17 and 8-9% for B73) than hyper DMRs (2.3-4.1%) were transmitted …" vs Page 15, Discussion: "1.1-1.5% hyper-DMR (TCM) and 5.2-7.0% hypo-DMR (TCdM) loci … can be transmitted".

2. It was known that the Mo17 genome is quite different the B73 genome (doi: 10.1104/pp.111.174748; 10.1038/s41588-018-0182-0) and extensive SVs exist across the genome. The possibility that some of the identified epialleles are due to SVs, for example copy number variation, should be considered/discussed.

3. Please explain why the mop1 hybrid was generated only in one direction. How that would affect interpretation of the results.

4. While the correlation between siRNA level and DNA methylation is nice and somewhat expected. Why do these loci tend to be transmitted? Are they correlated with certain types of TEs, sequences, or other epigenetic features? Do hypo tgDMRs have relatively lower sRNA levels compared to hyper tgDMRs? I think more analyses or discussion is needed to provide mechanistic implications.

Other minor points:

Page 6: Figure S1a should be S1b?

Figure S2b: This should be divided into two graphs, as not comparisons were not made between BM and MB lines.

Figure 3f, 4f, S4i: please explain how "Total" is calculated in the figure.

Figure S3: there were 2 e panels.

Please also double check all the citations of Dataset and supplemental figures.

**Reviewer 2**

**Are you able to assess all statistics in the manuscript, including the appropriateness of statistical tests used?**

No, I do not feel adequately qualified to assess the statistics.

**Comments to author:**

In this manuscript Cao et al. explore transgenerational inheritance of epialleles in maize and their connection to small RNAs. In detail, they explore DNA methylation across 6 backcross generations and 3 self-pollination generations of the hybridization between the inbred lines B73 and Mo17 in both directions. Their analysis identified several loci showing transgenerational inheritance that are connected to 24 nt sRNA accumulation and, accordingly, erased in the mop1-1 mutant, a component of the RNA directed DNA methylation (RdDM) pathway. Finally, they also correlate the identification of differentially methylation regions across generations with the expression of associated genes and their potential functions. The work is timely, well designed, with good quality data and most of their conclusions are well supported. Nevertheless, I have some comments that I hope will help to clarify some aspects of the manuscript. My first 4 comments could be considered as major although I think all of them are equally important to address.

1. It is confusing what the authors consider as a transgenerational DMR (tgDMR). This needs a major clarification since it is one of the major aspects analyzed on the manuscript. First, the authors comment that for the BM hybrid 7-10% of TCdM DMRs and 2-3% of TCM DMRs were tgDMRs (is "-" actually a "."?). Later in the text, it is commented that in the MB6S3 and the BM6S3 populations the number of tgDMRs were 19-60 and 19-35, respectively (hyper-hypo). Are those numbers referring to the number of tgDMRs that are conserved from the nonrecurrent parents or to real tgDMRs?

It would be more interesting and scientifically solid to classify as transgenerational only the DMRs that are truly inherited to the final populations MB6S3 and BM6S3, or at least use a different nomenclature for tgDMRs (the term epiallele is mentioned in the text but not consistently used and would be a good alternative) that are stable through all the crossing strategy.

2. The association of 24 nt sRNAs with hypermethylation is clear and has been well reported in different works, but this class of sRNAs is not connected at all with hypomethylation as the authors do in the manuscript. They do not make any distinction between hypo and hypermethylation and their connection with 24 nt sRNAs and simply use the term tgDMRs on their analyses. The authors need to address this aspect since this is another major component of their study. In brief, it cannot be justified that hypomethylated tgDMRs are associated with a higher presence of sRNAs. Are hypermethylation tgDMRs more associated with 24 nt sRNAs than hypomethylation tgDMRs? Is there any genomic characteristic (different histone mark presence, genomic feature, transcription, correlation with different sRNA classes, etc) different between hypo- and hypermethylated tgDMRs?

3. Are the phenotypes analyzed in Figure 5 lost or decreased in a mop1-1 mutant? Also, the connection between tgDMRs and gene expression changes should be analyzed in the mop1-1 mutant. These two experiments would be very convincing arguments for connecting the role of sRNAs in the inheritance of tgDMRs affecting gene expression.

4. The connection between the direction of the expression of epigenes (up- or downregulated) with tgDMRs (hypo- and hypermethylated) should be made. It is usually assumed that hypermethylation will induce downregulation of the associated gene while the opposite is expected from hypomethylation. Despite the authors showing a very convincing example, they fail to show the actual connection between DNA methylation and gene expression at the global level.

5. Changes in DNA methylation (or specifically CHH methylation) between the different lines analyzed using 100kb/bins should be tested statistically for significance of the differences indicated in the text.

6. Are tgDMRs conserved from nonrecurrent parents different from the rest of tgDMRs? A greater analysis of the genomic characteristics of this tgDMRs would be very interesting.

7. Global methylation values for the mop1-1 mutant should be shown somewhere in the main of supplementary figures (similar to the Kernel density analysis showed for the rest of the lines analyzed in the paper) and tested for statistical significance.

8. What are the characteristics of the TEs associated with tgDMRs? It would be interesting to know aspects of the biology of these TEs: family, length, age, transcription level, association with different sRNA classes and dependency of mop1-1 for the biogenesis of sRNAs.

Typos:

-Page 7, line 53 "hyer" should be "hyper".

-Page 8, line 47 "(2.3-4.1%)" should be (2-3% and 4-1%)? If not, please check the nomenclature of these percentages in the paragraph (7-10% and 2-3%).

-Page 13, line 31 "epiegenes" should be "epigenes".

**Authors Response**

**Point-by-point responses to the reviewers’ comments:**

Reviewer #1:

The manuscript titled "Small RNAs mediate transgenerational inheritance of genome-wide trans-acting epialleles in maize" by Chen et al. characterized the effect of hybridization on the DNA methylome in maize and followed the DNA methylome in backcrossing and selfing progenies. They identified hundreds of genomic loci that exhibited inheritance of hybrid-induced epigenetic changes (called tgDMRs). They also showed the correlation between sRNA levels and DNA methylation levels at these loci through small RNA sequencing. Importantly, mop1 hybrids were used to demonstrate that sRNA is necessary for hybrid-induced DNA methylation changes. In the end they examined the correlation between DNA methylation, sRNA and gene expression of selected genes. Transgenerational inheritance is clearly an important topic in epigenetics, and I think the experiment design is fine to answer the question(s) proposed. The overall logic of the manuscript is relatively easy to follow but main concerns exist, particularly on the presented DNA methylome data.

*Response: We thank this expert reviewer for succinct summary of our contribution.*

1. A clear definition of tgDMR is needed in the main text. I think the intuitive (and most strict) definition for tgDMRs are the ones that were identified as DMRs in all F1 and offspring generations. Considering the metastable nature of CHH methylation and measurement errors, this criterion can be slightly relaxed. However, I feel that multiple definitions of tgDMR were mixed in the text. A main table listing the number of DMRs should be included.

*Response: We are aware of the metastable nature of CHH methylation and statistical cut-off values (tests), which may affect the number of DMRs in each generation studied. Because of the variation among different generations, we used the DMRs that were present in the latest populations MB6S3 and BM6S3. Per suggestion, we generated Table 1 to list DMRs in all generations including F1 (TCdM and TCM), BM5 and MB5, MB6S1 and BM6S1, and MB6S3 and BM6S3 populations. The definition has been clarified. “As examples, 102 TCM (hyper) DMRs in the BM hybrid remained hyper DMRs in BM6S3 and also showed increased methylation levels in all other Mo17 backcross generations (Table 1), while 225 TCdM (hypo) DMRs remained hypo DMRs in BM6S3 with decreased methylation levels in all other backcross lines. These TCM and TCdM DMRs that were induced by hybridization and transmitted to the last backcross-selfing generation (BM6S3 or MB6S3) were called transgenerational DMRs (tgDMRs) or epialleles.” These numbers in Table 1 have also substantiated the changes in DMRs over different backcross generations in reciprocal directions. We thank this reviewer and reviewer #2 for the comment on the definition of DMRs and their inheritance patterns. A paragraph in the Methods was also added to clarify these points.*

a. Page 8, "a fraction of hybrid induced hyper- or hypo-DMR loci persisted over nine generations of backcrossing followed by selfing ……. These hyper (TCM) and hypo (TCdM) DMRs are called transgenerational DMRs (tgDMRs)". After reading the information from "Methods details", I'm still not clear whether these DMRs are "normal" DMRs (all contexts included) or CHH DMRs, or whether both criteria are satisfied as indciated in page 24.

*Response: As we replied above, these are all CHH DMRs. In fact, our study was focused on the CHH DMRs because CHG and CG methylation did not show obvious changes. “These analyses found substantial changes in CHH methylation levels (in 100-kb/bins) in the hybrids or backcross lines (Fig. 1b and Fig. S1, d-h), while CHG and CG methylation levels did not display obvious changes and could be used as internal controls. We focused our further analysis on changes in the CHH methylation context.”*

b. Assuming the 2 (both) criteria in page 24 were used for defining tgDMRs, numbers are inconsistent here and there in the main text.

*Response: We apologized for the ambiguity of tgDMR numbers and explained them as follows.*

Page 8: 327 and 462 tgDMRs in Mo17 and B73 backcross lines;

*Response: These are total tgDMRs in Mo17 and B73 backcross lines (Table 1).*

Page 9: 19 hyper tgDMRs and 60 hypo DMRs in MB6S3 population;

Page 9, paragraph 2: 19 hyper tgDMRs and 35 hypo tgDMRs in BM6S3 lines (whereas Figure S3 heatmaps indicates many more DMRs);

*Response: These are the tgDMRs each consist of SNPs that can be used to discriminate between B73 and Mo17 parental origins. These tgDMRs were used to indicate that tgDMRs were genetically homozygous to the recurrent parent in the last backcross-selfing generation. We clarified this in the revision. “Among all tgDMRs in BM6S3 and MB6S3 populations, there were allelic tgDMRs each with SNPs that could discriminate between B73 and Mo17 alleles, including 38 hyper (19 in MB6S3 and 19 in BM6S3) and 95 hypo (60 in MB6S3 and 35 in BM6S3) tgDMRs (Table 1, Fig. 2a, b, and Fig. S3f, h). Notably, all allelic tgDMRs with one exception were genetically homozygous to the recurrent parent in the last backcross-selfing generation. Only the locus (Chr9:141101601-141101800) had residual B73 sequence in BM6S3.”*

Page 9, last paragraph: 36.1% (118/327) and 31.0% (143/462) … were stably transmitted to the offspring through six backcrossing and three selfing generations (weren't all the tgDMRs stably transmitted? 118 and 143 are larger than 19+ 60 or 19 + 35?).

*Response: All tgDMRs could be potentially transmitted over generations. The purpose of this statement is to indicate the number of tgDMRs in BC6S3 that had CHH methylation levels similar to the nonrecurrent parent (origin). We revised the sentence, “Among tgDMRs in Mo17 and B73 backcross lines (Table S3), 36.1% (118/327) and 39.0% (182/462) that originated from their corresponding nonrecurrent parents in the F1 were transmitted to the BM6S3 and MB6S3 populations, respectively, through six backcrossing and three selfing generations (Table 1).”*

Page 8, paragraph 2: 7-10% TCdM DMRs in the BM hybrid were also hypo tgDMRs …, and 2-3% TCM DMRs in the BM hybrid also remained hyper tgDMRs … (it seems that tgDMRs here were used to indicate the same direction of methylation changes, instead of solely referring to the BM/MB6S3 generation).

*Response: These DMRs were transmitted to one of the Mo17 backcross generations (BM6S1 or BM6S3). In the revision, these DMRs were not called tgDMRs. Only DMRs transmitted to BC6S3 and showing corresponding DNA methylation changes in all backcross generations were defined as tgDMRs, including 327 tgDMRs in Mo17 backcross lines and 462 tgDRMs in B73 backcross lines. We revised, “Approximately 6-9% TCdM (hypo) DMRs and 2-4% TCM (hyper) DMRs in the BM hybrid were also identified as hypo DMRs (Fig. S3b) and hyper DMRs (Fig. S3c), respectively, in each of Mo17 backcross generation (BM6S1 or BM6S3). The same trend was observed in the B73 backcross lines, including 8-9% TCdM DMRs (Fig. S3d) and 2-3% TCM DMRs (Fig. S3e) in the MB hybrid, which remained hypomethylation and hypermethylation in respective B73 backcross generation (MB6S1 or MB6S3).”*

Page 8: "threefold or more hypo DMRs (7-10% for Mo17 and 8-9% for B73) than hyper DMRs (2.3-4.1%) were transmitted …" vs Page 15, Discussion: "1.1-1.5% hyper-DMR (TCM) and 5.2-7.0% hypo-DMR (TCdM) loci … can be transmitted".

*Response: In prior submission, ‘7-10% for Mo17 and 8-9% for B73’ indicates the DMRs transmitted in any one of the backcross generations. ‘1.1-1.5% hyper-DMR (TCM) and 5.2-7.0% hypo-DMR (TCdM) loci’ indicates the tgDMRs transmitted to the last backcross-selfing generation. In revision, we used the tgDMRs instead of these DMRs. “Interestingly, although TCM DMRs were twofold more abundant than TCdM DMRs (Fig. S2c), fivefold or more TCdM DMRs (5.5% for BM hybrid and 6.9% for MB hybrid) than TCM DMRs (1.5% for BM hybrid and 1.1% for MB hybrid) were transmitted in the Mo17 and B73 backcross populations, respectively.”*

2. It was known that the Mo17 genome is quite different the B73 genome (doi: 10.1104/pp.111.174748; 10.1038/s41588-018-0182-0) and extensive SVs exist across the genome. The possibility that some of the identified epialleles are due to SVs, for example copy number variation, should be considered/discussed.

*Response: We appreciated this comment and identified structural variation (SV) between B73 and Mo17 genomes (see Methods). To identify the SVs, Mo17 genome were divided into 10-kb windows with 100-bp steps (100x depth of genome), and then mapped onto B73 genome (RefGen_v4) using minimap2 software (v2.18-r1015) with default parameters [1]. Mapped results were sorted by Samtools to call SV using cuteSV (v1.0.11) with options “-s 10 –r 500 -l 50 -sl 50” [2]. A total of 175,849 SV were identified, including 94,779 DEL (deletion, 107,516,448 bp of B73-specific sequences, 59.8%), 77,705 INS (insertion, 62,043,121bp of Mo17-specific sequences, 34.5%), 2,784 DUP (duplications), 535 INV (inversion), and 46 TRL (translocation) (Fig. S4b). Approximately 2.1% (7/327) tgDMRs in the Mo17 backcross lines and 3.9% (18/462) tgDMRs in the B73 backcross lines overlapped with breakpoints of SV, which was higher than 1.3% (58,721/4,645,542) in whole genome (Fig. S4c).*

*In the revision, we included these in the Results. “To determine a possible contribution of structural variation (SV) to tgDMRs between B73 and Mo17 genomes, we identified 175,849 SV (Fig. S4b) and found that 2.1% (7/327) tgDMRs in Mo17 backcross lines and 3.9% (18/462) tgDMRs in B73 backcross lines overlapped with breakpoints of SV, which was higher than 1.3% (58,721/4,645,542) at the whole genome level (Fig S4c) (Chi-square test, P = 0). Furthermore, we found 2.1% (949/44,799) of these DMRs between B73 and Mo17 overlapped with the SV (Fig S4d), suggesting a contribution of SV to methylation level of flanking loci [3]. The tgDMRs with SV (7 in Mo17 backcross lines and 18 in B73 backcross lines) showed higher levels of methylation changes than tgDMRs without SV (Wilcoxon signed-rank test, P<1.8e-3) (Fig S4d). Although the total number of tgDMRs with SV was relatively small, these DMRs associated with SVs tended to be transgenerationally inherited.”*

3. Please explain why the mop1 hybrid was generated only in one direction. How that would affect interpretation of the results.

*Response: During the study, Mo17XB73:mop1 were generated and validated with a sufficient amount of seeds. In maize seedings, the difference between reciprocal crosses is often relatively small [4]. In Arabidopsis thaliana, the same set of 24nt siRNA and CHH methylation loci was lost between ColXC24:nrpd1/nrpe1 and C24XCol:nrpd1/nrpe1 [5] . In revision, we modified the statement. “We generated and validated Mo17XB73:mop1 hybrid to test a role of small RNAs and RdDM in tgDRMs during backcrossing. The reciprocal hybrid was not used for this study, which may miss some changes. However, in Arabidopsis the same set of 24-nt siRNA and CHH methylation loci was lost in the reciprocal crosses ColXC24:nrpd1/nrpe1 and C24XCol:nrpd1/nrpe1 [5], suggesting similar siRNA and RdDM changes in both directions in vegetative tissues (leaves), except in the seeds, as observed in Arabidopsis [6, 7].”*

4. While the correlation between siRNA level and DNA methylation is nice and somewhat expected. Why do these loci tend to be transmitted? Are they correlated with certain types of TEs, sequences, or other epigenetic features? Do hypo tgDMRs have relatively lower sRNA levels compared to hyper tgDMRs? I think more analyses or discussion is needed to provide mechanistic implications.

*Response: These tgDMRs were tended to locate in 5’ (12.8-15.3%) and 3’ (7.6-12.2%) regions flanking the coding sequence (Fig. S4a) (Chi-square test, P=0). Moreover, 34.5%-39.5% of these tgDMRs were associated with TEs, including class I and II TEs, with no indication of enrichment in specific TE families (Fig S7, a and b).*

*We also downloaded other datasets from NCBI (accession no. PRJNA381532, PRJNA527732), including Dnase-seq, ATAC-seq, and ChIP-seq of histone modifications (H3K27me3, H3K36me3, H3K56ac, H3K4me3, H3K4me1) to investigate chromatin status of tgDMRs. We found that these tgDMRs avoided open chromatin regions (Fig S4e-f). Moreover, these tgDMRs were depleted with H3K36me3, H3K56ac, H3K4me3, and H3K4me1 histone marks and H2A.Z (Fig. S4g). In contrast, H3K27me3, a repressive mark for gene expression, overlapped with these DMRs (Fig. S4g). These results indicate an association of tgDMRs with chromatin inaccessibility. We included this new set of data in the Results. “Using other epigenetic datasets, including Dnase-seq , ATAC-seq, and ChIP-seq of histone modifications (H3K27me3, H3K36me3, H3K56ac, H3K4me3, H3K4me1) [8] (NCBI accession no. PRJNA381532, PRJNA527732), we investigated chromatin status of tgDMRs. We found that these tgDMRs (327 in BM6S3 and 462 in MB6S3) were depleted with H3K36me3, H3K56ac, H3K4me3, and H3K4me1 histone marks and H2A.Z (Fig. S4g). In contrast, H3K27me3, a repressive mark for gene expression, overlapped with these DMRs (Fig. S4g). These results indicate an association of tgDMRs with chromatin inaccessibility.”*

*We found that changes of 24-nt siRNA expression levels were similar between hypo tgDMRs and hyper tgDMRs (Fig. S7, c and d), which was consistent with small RNA changes in TCM and TCdM DMRs of F1 hybrid in Arabidopsis [5]. In maize, these TCM and TCdM loci (including tgDMRs) were largely associated with TE-derived 24-nt siRNAs (Fig 3d, 3f, S3a, S3j and S5a). In the Discussion, we added, “Although the amount of siRNAs may not fully explain why there are more TCM than TCdM loci, these hybrid-induced DMRs are eliminated in the hybrids of mop1-1 mutants in maize, as in the nrpe1/nrpd1 mutant hybrids of Arabidopsis [5]. We also found some TCM and TCdM DMRs that were independent of the 24-nt siRNA of RdDM. Alternatively, these methylation epialleles can be maintained through other mechanisms such as RdDM-independent pathway [9, 10] and homeostasis of CHH methylation and transcriptional regulation of methylated TEs by PolIV [11].”*

Other minor points:

Page 6: Figure S1a should be S1b?

Figure S2b: This should be divided into two graphs, as not comparisons were not made between BM and MB lines.

Figure 3f, 4f, S4i: please explain how "Total" is calculated in the figure.

Figure S3: there were 2 e panels.

Please also double check all the citations of Dataset and supplemental figures.

*Response: As suggested, we corrected these and other errors and checked throughout the text. We fixed Fig. S2b and the labeling problem of 2 e panels in Fig. S3. “Total” represents siRNA expression from all windows of the whole genome.*

Reviewer #2:

In this manuscript Cao et al. explore transgenerational inheritance of epialleles in maize and their connection to small RNAs. In detail, they explore DNA methylation across 6 backcross generations and 3 self-pollination generations of the hybridization between the inbred lines B73 and Mo17 in both directions. Their analysis identified several loci showing transgenerational inheritance that are connected to 24 nt sRNA accumulation and, accordingly, erased in the mop1-1 mutant, a component of the RNA directed DNA methylation (RdDM) pathway. Finally, they also correlate the identification of differentially methylation regions across generations with the expression of associated genes and their potential functions. The work is timely, well designed, with good quality data and most of their conclusions are well supported. Nevertheless, I have some comments that I hope will help to clarify some aspects of the manuscript. My first 4 comments could be considered as major although I think all of them are equally important to address.

*Response: We appreciated positive comments by the expert reviewer and have addressed the comments as follows.*

1. It is confusing what the authors consider as a transgenerational DMR (tgDMR). This needs a major clarification since it is one of the major aspects analyzed on the manuscript. First, the authors comment that for the BM hybrid 7-10% of TCdM DMRs and 2-3% of TCM DMRs were tgDMRs (is "-" actually a "."?). Later in the text, it is commented that in the MB6S3 and the BM6S3 populations the number of tgDMRs were 19-60 and 19-35, respectively (hyper-hypo). Are those numbers referring to the number of tgDMRs that are conserved from the nonrecurrent parents or to real tgDMRs?

It would be more interesting and scientifically solid to classify as transgenerational only the DMRs that are truly inherited to the final populations MB6S3 and BM6S3, or at least use a different nomenclature for tgDMRs (the term epiallele is mentioned in the text but not consistently used and would be a good alternative) that are stable through all the crossing strategy.

*Response: Per suggestion by this reviewer and reviewer #1, we generated Table 1 to list DMRs in all generations, including F1 (TCdM and TCM), BM3 and MB3, BM5 and MB5, MB6S1 and BM6S1, and MB6S3, and BM6S3 populations. Transgenerational DMRs (tgDMRs) are defined as the TCM and TCdM DMRs that were induced by hybridization and transmitted to the last backcross-selfing generations (BM6S3 or MB6S3). These numbers in Table 1 have also substantiated the changes in DMRs over different backcross generations in reciprocal directions. We thank this reviewer and reviewer #1 for the comment related to the definition of DMRs and their inheritance patterns. We also included a paragraph in the Methods to clarify these points.*

*These DMRs were transmitted to one of Mo17 backcross generations (BM6S1 or BM6S3). In the revision, these DMRs were not called tgDMRs. Only DMRs transmitted to the BC6S3 and showing corresponding DNA methylation changes in all backcross generations were defined as tgDMRs, including 327 tgDMRs in Mo17 backcross lines and 462 tgDRMs in B73 backcross lines. We revised, “Approximately 6-9% TCdM (hypo) DMRs and 2-4% TCM (hyper) DMRs in the BM hybrid were also identified as hypo DMRs (Fig. S3b) and hyper DMRs (Fig. S3c), respectively, in each of Mo17 backcross generation (BM6S1 or BM6S3). The same trend was observed in the B73 backcross lines, including 8-9% TCdM DMRs (Fig. S3d) and 2-3% TCM DMRs (Fig. S3e) in the MB hybrid, which remained hypomethylation and hypermethylation in respective B73 backcross generation (MB6S1 or MB6S3).”*

“the number of tgDMRs were 19-60 and 19-35, respectively (hyper-hypo)”

*Response: These are the tgDMRs each consist of SNPs that can be used to discriminate between B73 and Mo17 parental origins. We clarified this in the revision. “Among all tgDMRs in BM6S3 and MB6S3 populations, subsets were allelic tgDMRs each with SNPs that could discriminate between B73 and Mo17 alleles, including 38 hyper (19 in MB6S3 and 19 in BM6S3) and 95 hypo (60 in MB6S3 and 35 in BM6S3) tgDMRs (Table 1, Fig. 2a, b, and Fig. S3f, h). Notably, these tgDMRs were genetically homozygous to the recurrent parent in the last backcross-selfing generation.”*

2. The association of 24 nt sRNAs with hypermethylation is clear and has been well reported in different works, but this class of sRNAs is not connected at all with hypomethylation as the authors do in the manuscript. They do not make any distinction between hypo and hypermethylation and their connection with 24 nt sRNAs and simply use the term tgDMRs on their analyses. The authors need to address this aspect since this is another major component of their study. In brief, it cannot be justified that hypomethylated tgDMRs are associated with a higher presence of sRNAs. Are hypermethylation tgDMRs more associated with 24 nt sRNAs than hypomethylation tgDMRs? Is there any genomic characteristic (different histone mark presence, genomic feature, transcription, correlation with different sRNA classes, etc) different between hypo- and hypermethylated tgDMRs?

*Response: This is a valid comment. We found that changes of 24-nt siRNA expression levels were similar between hypo tgDMRs and hyper tgDMRs (Fig. S7, c and d), which was consistent with small RNA changes in TCM and TCdM DMRs of F1 hybrid in Arabidopsis [5]. In maize, these TCM and TCdM loci (including tgDMRs) were largely associated with TE-derived 24-nt siRNAs (Fig 3d, 3f, S3a, S3j and S5a). In the Discussion, we added, “Although the amount of siRNAs may not fully explain why there are more TCM than TCdM loci, these hybrid-induced DMRs are eliminated in the hybrids of mop1-1 mutants in maize, as in the nrpe1/nrpd1 mutant hybrids of Arabidopsis [5]. We also found that some TCM and TCdM DMRs were independent of the 24-nt siRNA of RdDM. These epialleles can be maintained through other mechanisms such as RdDM-independent pathway [9, 10] and homeostasis of CHH methylation and transcriptional regulation of methylated TEs by PolIV [11].”*

*We also downloaded other datasets from NCBI (accession no. PRJNA381532, PRJNA527732), including Dnase-seq, ATAC-seq, and ChIP-seq of histone modification (H3K27me3, H3K36me3, H3K56ac, H3K4me3, H3K4me1) to investigate chromatin state of tgDMRs. We found that these tgDMRs avoided open chromatin regions (Fig S4e-f). Moreover, these tgDMRs were depleted of H3K36me3, H3K56ac, H3K4me3, and H3K4me1 histone marks and H2A.Z (Fig. S4g). In contrast, H3K27me3, a repressive mark for gene expression, overlapped with these DMRs (Fig. S4g). These results indicate an association of tgDMRs with chromatin inaccessibility. We included this new set of data in the Results. “These 327 and 462 tgDMRs in Mo17 and B73 backcross lines, respectively , were significantly enriched in 5’ (12.8-15.3%) and 3’ (7.6-12.2%) regions flanking the coding sequence (Fig S4a) (Chi-square test, P=0). Moreover, these tgDMRs were depleted of H3K36me3, H3K56ac, H3K4me3, and H3K4me1 histone marks and H2A.Z (Fig. S4g). In contrast, H3K27me3, a repressive mark for gene expression, overlapped with these DMRs (Fig. S4g). These results indicate an association of tgDMRs with chromatin inaccessibility but void of open chromatin sites.”*

3. Are the phenotypes analyzed in Figure 5 lost or decreased in a mop1-1 mutant? Also, the connection between tgDMRs and gene expression changes should be analyzed in the mop1-1 mutant. These two experiments would be very convincing arguments for connecting the role of sRNAs in the inheritance of tgDMRs affecting gene expression.

*Response: This is a valid comment. First, we measured seedling biomass at 9 DAP (Fig. S6b, c) of B73:mop1-1, Mo17:mop1-1 and Mo17XB73:mop1-1. We found that biomass were significantly decreased in all mop1-1 mutants compared to the respective wildtype, as well as in the hybrid Mo17XB73:mop1-1 compared to MPV (Fig. S6b, c). The result suggests a role of methylation loss (mop1-1 mutant) in seedling growth and biomass heterosis.*

*Second, due to similar phenotypic alteration from loss of 24-nt RNAs in the mop1-1 mutant in different genetic backgrounds (Fig S5, b-d), we used B73:mop1-1 to verify the role of siRNAs in the contribution of tgDMRs to gene expression. Five of epigenes associated with tgDMRs of B73 backcross lines were randomly selected for qRT-PCR analysis of expression. We found that 4 of 5 (80%) tested were significantly upregulated, and the other one (Zm00001d033278) had the same trend of upregulation in the B73:mop1-1 mutant (Fig. S6d). These results were included in the revision. “The loss of small RNAs in the mop1-1 mutant has a negative effect on seedling growth and biomass heterosis. We found seedling biomass of mop1-1 mutants was significantly reduced relative to the wildtype lines (Fig. S6, b and c). Moreover, seedling biomass heterosis was also decreased in the hybrid Mo17XB73:mop1-1. The correlation of mop1-1 mutant with the reduced level of seedling growth was consistent with gene expression data. Among five epigenes that were associated with tgDMRs and randomly selected for quantitative RT-PCR analysis (Table S4), we found that four of five (80%) epigenes tested were significantly upregulated, and the other one (Zm00001d033278) had the same trend of upregulation in the B73:mop1-1 mutant (Fig. S6d).”*

4. The connection between the direction of the expression of epigenes (up- or downregulated) with tgDMRs (hypo- and hypermethylated) should be made. It is usually assumed that hypermethylation will induce downregulation of the associated gene while the opposite is expected from hypomethylation. Despite the authors showing a very convincing example, they fail to show the actual connection between DNA methylation and gene expression at the global level.

*Response: This comment is interesting, as the correlation of epigenes with methylation changes may not be straightforward, positive or negative depending on the genes (housing keeping or otherwise) and location of methylation (5’ or 3’ or gene body) [12-14]. At the genome-wide level, we found weak correlation between DNA methylation and expression levels of the epigenes associated with tgDMRs as shown in Fig. 5d, c and also in Fig. R1, a and b (P = 0.2;), despite four of five epigenes were upregulated (Fig. S6d). In addition, when hyper DMRs was related to a repressor of gene expression, high methylation levels can promote expression of epigenes, as observed for upregulation of ROS1 expression in sensing methlation homeotsis [15] and in Arabidopsis allotetraploids [16]. At the genome-wide level, there was a trend of elevated expression of epigenes compared to all of genes. Epigenes displayed significantly higher expression fold changes than all genes (P< 3.95e-9 in B73 backcross lines and P< 1.28e-8 in Mo17 backcross lines, Wilcoxon signed-rank test) (Fig. S7h). Thess results suggest that methylation changes in the tgDMRs may alter binding affinity of activators or repressors to alter epigene expression, as recently reported [17]. We included these views in the revision.*

5. Changes in DNA methylation (or specifically CHH methylation) between the different lines analyzed using 100kb/bins should be tested statistically for significance of the differences indicated in the text.

*Response: Per suggestion, we added statistical tests for Fig. S1d-h.*

6. Are tgDMRs conserved from nonrecurrent parents different from the rest of tgDMRs? A greater analysis of the genomic characteristics of this tgDMRs would be very interesting.

*Response: Per suggestion, we analzyed the data and found similar genomic features (Fig R2a), chromatin accessibility (Fig R2b), and histone modifications (Fig R2c) of the tgDMRs conserved from nonrecurrent parents and the rest of tgDMRs.*

7. Global methylation values for the mop1-1 mutant should be shown somewhere in the main of supplementary figures (similar to the Kernel density analysis showed for the rest of the lines analyzed in the paper) and tested for statistical significance.

*Response: This is a good suggestion. We added statistical tests for genome-wide methylation values in MB:mop1-1 hybrids and mutants (Fig. S5h, i, k, l) (P = 0, Wilcoxon signed-rank test).*

8. What are the characteristics of the TEs associated with tgDMRs? It would be interesting to know aspects of the biology of these TEs: family, length, age, transcription level, association with different sRNA classes and dependency of mop1-1 for the biogenesis of sRNAs.

*Response: These tgDMRs were tended to locate in 5’ (12.8-15.3%) and 3’ (7.6-12.2%) regions flanking the coding sequence (Fig. S4a) (P=0, Chi-square test). Approximately 34.5%-39.5% of these tgDMRs were associated with TEs, including class I and II TEs, with no clear enrichment of specific TE families (Fig. S7, a and b). However, these tgDMRs were not enriched among specific TE families (Fig. S7, a and b), but were associated with longer TEs (P < 1.2e-4 , Wilcoxon signed-rank test) (Fig. S7c), LTRs with older insertion age (P < 1.8e-3 , Wilcoxon signed-rank test) (Fig. S7d), and TEs with higher expression levels of 24-nt siRNAs (P< 1.4e-3 , Wilcoxon signed-rank test) (Fig. S7e).”*

Typos:

-Page 7, line 53 "hyer" should be "hyper".

-Page 8, line 47 "(2.3-4.1%)" should be (2-3% and 4-1%)? If not, please check the nomenclature of these percentages in the paragraph (7-10% and 2-3%).

-Page 13, line 31 "epiegenes" should be "epigenes".

*Response: Per suggestion, we have corrected these typos and several others in the text. For p. 8 line 47, we clarified these numbers in the revision following the “revised” definition of tgDMRs.*

*References cited in the response letter*

*1. Li H: Minimap2: pairwise alignment for nucleotide sequences. Bioinformatics 2018, 34(18):3094-3100.*

*2. Jiang T, Liu Y, Jiang Y, Li J, Gao Y, Cui Z, Liu Y, Liu B, Wang Y: Long-read-based human genomic structural variation detection with cuteSV. Genome Biol 2020, 21(1):189.*

*3. Springer NM, Anderson SN, Andorf CM, Ahern KR, Bai F, Barad O, Barbazuk WB, Bass HW, Baruch K, Ben-Zvi G et al: The maize W22 genome provides a foundation for functional genomics and transposon biology. Nat Genet 2018, 50(9):1282-1288.*

*4. Ko DK, Rohozinski D, Song Q, Taylor SH, Juenger TE, Harmon FG, Chen ZJ: Temporal shift of circadian-mediated gene expression and carbon fixation contributes to biomass heterosis in maize hybrids. PLoS Genet 2016, 12(7):e1006197.*

*5. Zhang Q, Wang D, Lang Z, He L, Yang L, Zeng L, Li Y, Zhao C, Huang H, Zhang H et al: Methylation interactions in Arabidopsis hybrids require RNA-directed DNA methylation and are influenced by genetic variation. Proc Natl Acad Sci U S A 2016, 113(29):E4248-4256.*

*6. Kirkbride RC, Lu J, Zhang C, Mosher RA, Baulcombe DC, Chen ZJ: Maternal small RNAs mediate spatial-temporal regulation of gene expression, imprinting, and seed development in Arabidopsis. Proc Natl Acad Sci U S A 2019, 116(7):2761-2766.*

*7. Lu J, Zhang C, Baulcombe DC, Chen ZJ: Maternal siRNAs as regulators of parental genome imbalance and gene expression in endosperm of Arabidopsis seeds. Proc Natl Acad Sci USA 2012, 109(14):5529-5534.*

*8. Mendieta JP, Marand AP, Ricci WA, Zhang X, Schmitz RJ: Leveraging histone modifications to improve genome annotations. G3 (Bethesda) 2021, 11(10).*

*9. Song Q, Guan X, Chen ZJ: Dynamic Roles for Small RNAs and DNA Methylation during Ovule and Fiber Development in Allotetraploid Cotton. PLoS Genet 2015, 11(12):e1005724.*

*10. Zemach A, Kim MY, Hsieh PH, Coleman-Derr D, Eshed-Williams L, Thao K, Harmer SL, Zilberman D: The Arabidopsis Nucleosome Remodeler DDM1 Allows DNA Methyltransferases to Access H1-Containing Heterochromatin. Cell 2013, 153(1):193- 205.*

*11. Zhang H, Lang Z, Zhu JK: Dynamics and function of DNA methylation in plants. Nat Rev Mol Cell Biol 2018, 19(8):489-506.*

*12. Zhang X, Yazaki J, Sundaresan A, Cokus S, Chan SW, Chen H, Henderson IR, Shinn P, Pellegrini M, Jacobsen SE et al: Genome-wide high-resolution mapping and functional analysis of DNA methylation in Arabidopsis. Cell 2006, 126(6):1189-1201.*

*13. Gent JI, Ellis NA, Guo L, Harkess AE, Yao Y, Zhang X, Dawe RK: CHH islands: de novo DNA methylation in near-gene chromatin regulation in maize. Genome Res 2013, 23(4):628-637.*

*14. Wang L, Cao S, Wang P, Lu K, Song Q, Zhao FJ, Chen ZJ: DNA hypomethylation in tetraploid rice potentiates stress-responsive gene expression for salt tolerance. Proc Natl Acad Sci U S A 2021, 118(13):e2023981118.*

*15. Lei M, Zhang H, Julian R, Tang K, Xie S, Zhu JK: Regulatory link between DNA methylation and active demethylation in Arabidopsis. Proc Natl Acad Sci U S A 2015, 112(11):3553-3557.*

*16. Jiang X, Song Q, Ye W, Chen ZJ: Concerted genomic and epigenomic changes accompany stabilization of Arabidopsis allopolyploids. Nat Ecol Evol 2021.*

*17. Han T, Wang F, Song Q, Ye W, Liu T, Wang L, Chen ZJ: An epigenetic basis of inbreeding depression in maize. Sci Adv 2021:(in press).*

**Second round of review**

**Reviewer #1:**

The authors have improved the manuscript by performing additional analyses and adding clearer definitions for tgDMRs and epialleles. I think most of concerns have been addressed. However, I do have following questions to clarify some issues.

1. Table 1, last column. What are "tgDMRs resembled the nonrecurrent parent"?

2. On the correlation between tgDMRs and other genomic features (Fig. S4). The authors showed that there is positive correlation of tgDMRs with H3K27me3, and negative correlation with active marks including H3K36me3, H3K56ac, H3K4me3, H3K4me1. Do hyper- and hypo-DMRs exhibit similar patterns? A similar question can be raised on the correlation between tgDMRs and siRNA levels (Fig. 3f and S5j). Were high-level 24-nt siRNAs detected on hypo tgDMRs as well?

3. Page 7, line 7. "the DMRs induced in the F1 hybrids". Which DMRs are these? Are they are TCM and TCdM DMRs defined in the next paragraph?

4. Page 9, paragraph 1. "TCM DMRs were twofold more abundant than TCdM DMRs", from which generation these DMRs refer to? "fivefold or more TCdM DMRs … were transmitted …" 5.5% divided by 1.5% equals 3.6. How was the fold change calculated?

5. Page 11, line 8. "higher than 1.0% (44,798/4,645,542) at the whole genome level". It is not clear where the numbers 44,798/4,645,542 are from.

6. Page 16, line 3 from bottom. This line repeats the meaning of the previous sentence.

**Reviewer #2:**

The authors have addressed most of my comments. Nevertheless, there are still a couple of aspects that should be clarified:

1. As shown in their response there is a lack a correlation between 24-nt siRNAs and hyper- or hypomethylation. Is there any other sRNA size-class that might help to understand the difference between accumulation of 24-nt siRNAs from a region that is recalcitrant to DNA methylation? How is it justified that a hypomethylated tgDMR accumulates 24-nt siRNAs? I think that the authors should give a reasonable explanation for this fact. If not, I don't think that the idea shown in the paper, about sRNAs driving tgDMRs, is fully supported. Additionally, the authors found that tgDMRs are enriched in H3K27me3 a histone mark that repels DNA methylation, this fact should be mentioned in the discussion as an alternative regulatory mechanism for these regions. In my opinion all these pieces of evidence point to multiple mechanisms regulating tgDMRs. This fact should be discussed at least in the discussion section of the manuscript.

2. The authors should clarify in the text what are the percentages separated by a bar in page 8 line 20 (6-9% and 2-4%), page 9 line 14 (12.8-15.3% and 7.6-12.2%), page 11 line 36 (9-15% and 6-11%) are referring to. This is not straightforward for the reader.

3. Typos:

-page 4: "parentis" should be "parent"

-page 15: "methlation homeostasis" should be "methylation homeostasis"

-page 15: "Thess" should be "These"

**Authors Response**

**Point-by-point responses to the reviewers’ comments:**

Reviewer #1:

The authors have improved the manuscript by performing additional analyses and adding clearer definitions for tgDMRs and epialleles. I think most of concerns have been addressed. However, I do have following questions to clarify some issues.

1. Table 1, last column. What are "tgDMRs resembled the nonrecurrent parent"?

*Response: We thank this expert reviewer for constructive comments that helped us improve the manuscript. We clarified the meaning of “resembled,” “methylation levels of these tgDMRs in a backcross line were similar to that of its nonrecurrent parent.”*

2. On the correlation between tgDMRs and other genomic features (Fig. S4). The authors showed that there is positive correlation of tgDMRs with H3K27me3, and negative correlation with active marks including H3K36me3, H3K56ac, H3K4me3, H3K4me1. Do hyper- and hypo-DMRs exhibit similar patterns? A similar question can be raised on the correlation between tgDMRs and siRNA levels (Fig. 3f and S5j). Were high-level 24-nt siRNAs detected on hypo tgDMRs as well?

*Response: This is a valid comment. As shown in Fig. S4g, both hyper and hypo DMRs show similar patterns, but the levels were much lower in hypo DMRs than in hyper DMRs. Consistently, levels of 24-nt siRNAs and 21/22-nt siRNAs were also higher in hyper DMRs than in hypo DMRs (Fig. S4e).*

*In the Discussion, we added, “Thus, RdDM pathway may account for hyper DMRs induced by hybridization. For hypo DMRs, low levels of 24-nt and/or 21/22-nt siRNAs may not sufficiently maintain RdDM (Greaves et al., 2016; Nuthikattu et al., 2013), which may involve other mechanisms such as RdDM-independent pathway (Song et al., 2015; Zemach et al., 2013), homeostasis of CHH methylation and transcriptional regulation of methylated TEs by Pol IV (Zhang et al., 2018), and H3K27me3 histone modifications for non-CG methylation (Zhou et al., 2016). For example, 37% of H3K27me3-marked genes are also methylated at non-CG sites in rice (Zhou et al., 2016). They may form feedback loops, and SDG711 can catalyze H3K27me3 modification via interaction with OsDRM2 for CHH methylation.”*

3. Page 7, line 7. "the DMRs induced in the F1 hybrids". Which DMRs are these? Are they are TCM and TCdM DMRs defined in the next paragraph?

*Response: Yes. Hybridization can induce both hyper and hypo DMRs or TCM and TCdM DMRs. These TCM and TCdM DMRs were used to emphasize trans-acting effects.*

4. Page 9, paragraph 1. "TCM DMRs were twofold more abundant than TCdM DMRs", from which generation these DMRs refer to? "fivefold or more TCdM DMRs … were transmitted …" 5.5% divided by 1.5% equals 3.6. How was the fold change calculated?

*Response: Thank you for pointing this out. It was an error, and the fold should be 3.6. We revised the sentence, “…although TCM DMRs (6,757 in the MB hybrid and 8,723 in the BM hybrid) were higher than TCdM DMRs (4,037 in the BM hybrid, and 5,212 in the MB hybrid) (Fig. S2c), 3-6-fold or more TCdM DMRs than TCM DMRs (5.5% vs. 1.5% in the BM hybrid and 6.9% vs. 1.1% in the MB hybrid) were transmitted in the Mo17 and B73 backcrossselfing populations, respectively…”*

5. Page 11, line 8. "higher than 1.0% (44,798/4,645,542) at the whole genome level". It is not clear where the numbers 44,798/4,645,542 are from.

*Response: We divided the whole genome into 4,645,542 bins used to identify DMRs through pairwise comparison, and 44,798 of them were DMRs between B73 and Mo17. Among these DMRs, 36-39% were tgDMRs, which were higher than ~1% (44,798 DMRs out of 4,645,542 total genomic bins) in the whole genome level.*

6. Page 16, line 3 from bottom. This line repeats the meaning of the previous sentence.

*Response: We have deleted the sentence to avoid repetition.*

Reviewer #2:

The authors have addressed most of my comments. Nevertheless, there are still a couple of aspects that should be clarified:

1. As shown in their response there is a lack a correlation between 24-nt siRNAs and hyper- or hypomethylation. Is there any other sRNA size-class that might help to understand the difference between accumulation of 24-nt siRNAs from a region that is recalcitrant to DNA methylation? How is it justified that a hypomethylated tgDMR accumulates 24-nt siRNAs? I think that the authors should give a reasonable explanation for this fact. If not, I don't think that the idea shown in the paper, about sRNAs driving tgDMRs, is fully supported. Additionally, the authors found that tgDMRs are enriched in H3K27me3 a histone mark that repels DNA methylation, this fact should be mentioned in the discussion as an alternative regulatory mechanism for these regions. In my opinion all these pieces of evidence point to multiple mechanisms regulating tgDMRs. This fact should be discussed at least in the discussion section of the manuscript.

*Response: With respect to 24-nt siRNA levels, we found that they higher in hyper DMRs than*

*in hypo DMRs, which were higher than in the random genomic regions. However, these 24-*

*nt siRNAs in hypo tgDMRs may be lower than below the threshold levels that are required to*

*maintain the RdDM pathway (Greaves et al., 2016). This may involve other epigenetic modifications to establish and maintain hypo DMRs (see below).*

*We agreed and included the following statement in the revision. This does not preclude that other siRNAs such as 21/22-nt siRNAs may also be involved (Nuthikattu et al., 2013). Indeed, distribution patterns of 21/22-nt siRNAs are consistent with those of 24-nt siRNAs (Fig. S4e), as these noncanonical siRNAs may involve silencing of TEs (Nuthikattu et al., 2013).*

*With respect to H3K27me3 repressive mark, it is mutually exclusive with CG methylation but not with non-CG (CHH and CHG) methylation. For example, 37% of H3K27me3-marked genes are also methylated at non-CG sites in rice (Zhou et al., 2016). They may form feedback loops, and SDG711 can catalyze H3K27me3 modification via interaction with OsDRM2 for CHH methylation (Zhou et al., 2016). In the Discussion, we added, “Thus, RdDM pathway may account for hyper DMRs induced by hybridization. For hypo DMRs, low levels of 24-nt and/or 21/22-nt siRNAs may not sufficiently maintain RdDM (Greaves et al., 2016; Nuthikattu et al., 2013), which may involve other mechanisms such as RdDM-independent pathway (Song et al., 2015; Zemach et al., 2013), homeostasis of CHH methylation and transcriptional regulation of methylated TEs by Pol IV (Zhang et al., 2018), and H3K27me3 histone modifications for non-CG methylation (Zhou et al., 2016). For example, 37% of H3K27me3-marked genes are also methylated at non-CG sites in rice (Zhou et al., 2016). They may form feedback loops, and SDG711 can catalyze H3K27me3 modification via interaction with OsDRM2 for CHH methylation.”*

*“The involvement of RdDM independent pathway may also explain why more TCdM than TCM loci tend to be transgenerational, since the amount of siRNAs may vary or dilute during selfing and backcross generations, as few siRNAs can transmit from sperm to the zygote in Arabidopsis (Kirkbride et al., 2019; Walker et al., 2018). Among these trans-acting methylation loci, 5.5-6.9% hypo-DMR (TCdM) and 1.1-1.5% hyper-DMR (TCM) loci in the reciprocal hybrids can be transmitted through multiple backcross and selfing generations.”*

2. The authors should clarify in the text what are the percentages separated by a bar in page 8 line 20 (6-9% and 2-4%), page 9 line 14 (12.8-15.3% and 7.6-12.2%), page 11 line 36 (9-15% and 6-11%) are referring to. This is not straightforward for the reader.

*Response: Thank you. We have revised these sentences as below.*

*On page 8 line 20, “Approximately ~7.5% TCdM (hypo) DMRs and ~2.4% TCM (hyper) DMRs in the BM hybrid were also identified as hypo DMRs (Fig. S3b) and hyper DMRs (Fig. S3c), respectively, in each of Mo17 backcross generation (BM3 to BM6S3). The same trend was observed in the B73 backcross lines, including ~11% TCdM DMRs (Fig. S3d) and ~2.8% TCM DMRs (Fig. S3e) in the MB hybrid, which remained hypo- and hypermethylation in respective B73 backcross generation (MB3 to MB6S3).”*

*On page 9 line 14, “These 327 and 462 tgDMRs in Mo17 and B73 backcross lines, respectively (Table 1), were significantly enriched in 5’ (12.8% and 15.3% in Mo17 and B73 backcross lines, respectively) and 3’ (12.2% and 7.6% in Mo17 and B73 backcross lines, respectively) regions flanking the coding sequence (Fig S4a) (P = 0, Chi-square test).”*

*On page 11 line 36, “they were located more in the 5’ (15% and 9% in BM and MB hybrids, respectively ) and 3’ (11% and 6% in BM and MB hybrids, respectively) regions flanking the coding sequence than the genome-wide average (Fig. 3a).”*

3. Typos:

-page 4: "parentis" should be "parent"

-page 15: "methlation homeostasis" should be "methylation homeostasis"

-page 15: "Thess" should be "These"

*Response: As suggested, we corrected these errors and others.*

*References cited*

*Greaves, I. K., Eichten, S. R., Groszmann, M., Wang, A., Ying, H., Peacock, W. J., and Dennis, E. S. (2016). Twenty-four-nucleotide siRNAs produce heritable trans-chromosomal methylation in F1 Arabidopsis hybrids. Proc Natl Acad Sci U S A 113, E6895-E6902.*

*Kirkbride, R. C., Lu, J., Zhang, C., Mosher, R. A., Baulcombe, D. C., and Chen, Z. J. (2019). Maternal small RNAs mediate spatial-temporal regulation of gene expression, imprinting, and seed development in Arabidopsis. Proc Natl Acad Sci U S A 116, 2761- 2766.*

*Nuthikattu, S., McCue, A. D., Panda, K., Fultz, D., DeFraia, C., Thomas, E. N., and Slotkin, R. K. (2013). The initiation of epigenetic silencing of active transposable elements is triggered by RDR6 and 21-22 nucleotide small interfering RNAs. Plant Physiol 162, 116-31.*

*Song, Q., Guan, X., and Chen, Z. J. (2015). Dynamic Roles for Small RNAs and DNA Methylation during Ovule and Fiber Development in Allotetraploid Cotton. PLoS Genet 11, e1005724.*

*Walker, J., Gao, H., Zhang, J., Aldridge, B., Vickers, M., Higgins, J. D., and Feng, X. (2018). Sexual-lineage-specific DNA methylation regulates meiosis in Arabidopsis. Nat Genet 50, 130-137.*

*Zemach, A., Kim, M. Y., Hsieh, P. H., Coleman-Derr, D., Eshed-Williams, L., Thao, K., Harmer, S. L., and Zilberman, D. (2013). The Arabidopsis Nucleosome Remodeler DDM1 Allows DNA Methyltransferases to Access H1-Containing Heterochromatin. Cell 153, 193- 205.*

*Zhang, H., Lang, Z., and Zhu, J. K. (2018). Dynamics and function of DNA methylation in plants. Nat Rev Mol Cell Biol 19, 489-506.*

*Zhou, S., Liu, X., Zhou, C., Zhou, Q., Zhao, Y., Li, G., and Zhou, D. X. (2016). Cooperation between the H3K27me3 Chromatin Mark and Non-CG Methylation in Epigenetic Regulation. Plant Physiol 172, 1131-1141.*
